# Supplementary material for: Adenosine-Mimicking Derivatives of 3-Aminopyrazine-2-Carboxamide: Towards Inhibitors of Prolyl-tRNA Synthetase with Antimycobacterial Activity
Source: Biomolecules. 2022 Oct 26;12(11):1561. doi: 10.3390/biom12111561 (PMC9687610; doi:10.3390/biom12111561)
Supplement: Supplementary file 1 [file biomolecules-12-01561-s001.zip › biomolecules-1962829-supplementary.pdf]

Adenosine-mimicking derivatives of 3-aminopyrazine-2-carboxamide: towards inhibitors of prolyl-tRNA synthetase with antimycobacterial activity

Pallabothula V.S.K.[1], Kerda M.[1], Juhás M.[1], Janďourek O.[1], Konečná K.[1], Bárta P.[1], Paterová P.[2], Zitko J.[1]

[1] Faculty of Pharmacy in Hradec Králové, Charles University, Ak. Heyrovského 1203/8, 500 05 Hradec Králové, Czech Republic

[2] University Hospital Hradec Králové, Department of Clinical Microbiology, Sokolská 581, 500 05 Hradec Králové, Czech Republic

SUPPLEMENTARY MATERIAL

1. Methods 2
- 1.1 Antimicrobial screening 2
  - 1.1.1. *In vitro* screening of antimycobacterial activity against *Mycobacterium tuberculosis* H37Ra, *M. smegmatis*, *M. aurum* 2
  - 1.1.2. *In vitro* screening of antimycobacterial activity against *Mycobacterium tuberculosis* H37Rv and multidrug-resistant (MDR) strains of *Mtb* 3
  - 1.1.3. *In vitro* antibacterial evaluation 4
  - 1.1.4. *In vitro* antifungal evaluation 5
- 1.2. *In vitro* HepG2 cytotoxicity determination 7

2. Additional results 8
- 2.1. Analytical data 8
- 2.2. Representative NMR spectra 20
- 2.3. Results of antimycobacterial activity screening 25
- 2.4. Results of antibacterial activity screening 26
- 2.5. Results of antifungal screening 27
- 2.6. Results for HepG2 cytotoxicity 29
- 2.7. Results of *in silico* simulations 31

3. References 36

1. Methods

1.1 Antimicrobial screening

1.1.1. *In vitro* screening of antimycobacterial activity against *Mycobacterium tuberculosis* H37Ra, *M. smegmatis*, *M. aurum*

Microdilution assay (Microplate Alamar Blue Assay (MABA)) was performed as described in our earlier publications. The antimycobacterial assay was performed with fast-growing *Mycobacterium smegmatis* DSM 43465 (ATCC 607) and *M. aurum* DSM 43999 (ATCC 23366) from the German Collection of Microorganisms and Cell Cultures (Braunschweig, Germany) and with the avirulent strain of *Mycobacterium tuberculosis* H37Ra ITM-M006710 (ATCC 9431) from Belgian Coordinated Collections of Microorganisms (Antwerp, Belgium). The technique used for activity determination was the microdilution broth method using 96-well microtitration plates. The culturing medium was Middlebrook 7H9 broth (Sigma-Aldrich) with a defined pH of 6.6 ± 0.2 enriched with glycerol (Sigma-Aldrich) and Middlebrook OADC growth supplement (Himedia, Mumbai, India) according to the manufacturer's instructions.

The mycobacterial strains were cultured on enriched Middlebrook 7H9 agar, and suspensions were prepared in enriched Middlebrook 7H9 broth. The final density was adjusted to value 1.0 according to McFarland scale and diluted in the ratio 1:20 (for fast-growing mycobacteria) or 1:10 (for *Mtb* H37Ra) with broth.

The tested compounds were dissolved in dimethyl sulfoxide (DMSO) (Sigma-Aldrich), then Middlebrook 7H9 broth was added to obtain a concentration of 2000 µg/mL. The standards used for activity determination were isoniazid (INH), rifampicin (RIF), and ciprofloxacin (CIP) (Sigma-Aldrich). The final concentrations were reached by binary dilution and the addition of mycobacterial suspension and were set as 500, 250, 125, 62.5, 31.25, 15.625, 7.81, 3.91, and 1.95 µg/mL. The final concentration of DMSO in any well did not exceed 2.5% (*v*/*v*) and did not affect the growth of mycobacteria. Positive (broth, DMSO, bacteria) and negative (broth, DMSO) growth controls were included.

The plates were incubated in the dark at 37 °C without agitation. The addition of 0.01% solution of resazurin sodium salt followed after 48 h of incubation for *M. smegmatis*, after 72 h of incubation for *M. aurum,* and after 120 h of incubation for *Mtb* H37Ra, respectively. The stain was prepared by dissolving resazurin sodium salt (Sigma-Aldrich) in deionised water to get a 0.02% solution. Then, a 10% aqueous solution of Tween 80 (Sigma-Aldrich) was prepared. Both liquids were mixed up making use of the same volumes and filtered through a 0.2 µm syringe membrane filter. The microtitration panels were then incubated for further 2.5 h for the determination of activity against *M. smegmatis*, 4 h for *M. aurum*, and 18 h for *Mtb* H37Ra, respectively.

The antimycobacterial activity was expressed as the minimum inhibitory concentration (MIC). The MIC (in µg/mL) was determined based on stain colour change (blue colour—active; pink colour—not active). The MIC values for standards are presented in the respective tables. All experiments were conducted in duplicate.

1.1.2. *In vitro* screening of antimycobacterial activity against *Mycobacterium tuberculosis* H37Rv and multidrug-resistant (MDR) strains of *Mtb*

Microdilution method based on Microplate Alamar Blue Assay (MABA) was applied. Tested strain *Mycobacterium tuberculosis* H37Rv CNCTC My 331/88 (ATCC 27294) was obtained from the Czech National Collection of Type Cultures (CNCTC), National Institute of Public Health (Prague, Czech Republic). Multidrug-resistant strains of *Mtb* laboratory ID designation IZAK and MATI were obtained from the Department of Clinical Microbiology, University Hospital Hradec Králové from Dr. Pavla Paterová. Middlebrook 7H9 broth of declared pH 6.6 (Sigma-Aldrich) enriched with 0.5% of glycerol (Sigma-Aldrich) and 10% of OADC growth supplement (Himedia, Mumbai, India) was used for cultivation.

Tested compounds were dissolved and diluted in DMSO and mixed with broth (25 µL of DMSO solution in 2.475 mL of broth) and placed (100 µL) into microplate wells. Mycobacterial inocula were suspended in isotonic saline solution and the density was adjusted to 0.5–1.0 according to McFarland scale. These suspensions were diluted by 10^{-1} and used to inoculate the testing wells, adding 100 µL of mycobacterial suspension per well. The final concentrations of tested compounds in wells were 100, 50, 25, 12.5, 6.25, 3.13 and 1.56 µg/mL. INH was used as a standard (inhibition of growth). Positive control (visible growth) consisted of broth plus mycobacterial suspension plus DMSO. A total of 30 µL of Alamar Blue working solution (1:1 mixture of 0.02% resazurin sodium salt (aq. sol.) and 10% Tween 80) was added after five days of incubation. Results were then determined after 24 h of incubation. The MIC (in µg/mL) was determined as the lowest concentration that prevented the blue-to-pink colour change. All experiments were conducted in duplicates.

Susceptibility profiles for the used MDR *Mtb* strains

Mtb laboratory ID IZAK, isolated from a 63-years-old man from bronchial aspirate in 2020, tested and interpreted according to CLSI (Clinical and Laboratory Standards Institute) breakpoints in 2020. *Mtb* laboratory ID MATI, isolated from a 23-years-old man from sputum in 2021, tested and interpreted according to CLSI breakpoints in 2021. Drug susceptibility is summarised in Table S1.

Table S1 *Susceptibility profiles for the used MDR Mtb strains*

Laboratory ID	Drug	Minimum inhibitory concentration (μg/ml)	Susceptibility
IZAK	STM	4	resistant
	INH	4	resistant
	RIF	>8	resistant
	EMB	0.5	sensitive
	PZA	>16	resistant
MATI	STM	>16	resistant
	INH	>8	resistant
	RIF	>8	resistant
	EMB	0.5	sensitive
	PZA	>128	resistant

STM – streptomycin, INH – isoniazid, RIF – rifampicin, EMB – ethambutol, PZA – pyrazinamide

1.1.3. *In vitro* antibacterial evaluation

The microdilution broth method was performed according to EUCAST (The European Committee on Antimicrobial Susceptibility Testing) instructions with slight modifications.[1] Eight tested bacterial strains were purchased from the Czech Collection of Microorganisms (CCM, Brno, Czech Republic) or from the German Collection of Microorganisms and Cell Cultures (DSM, Braunschweig, Germany): *Staphylococcus aureus* subsp. *aureus* CCM 4223 (ATCC 29213), *Staphylococcus aureus* subsp. *aureus* methicillin-resistant (MRSA) CCM 4750 (ATCC 43300), *Staphylococcus epidermidis* CCM 4418 (ATCC 12228), *Enterococcus faecalis* CCM 4224 (ATCC 29212), *Escherichia coli* CCM 3954 (ATCC 25922), *Klebsiella pneumoniae* CCM 4415 (ATCC 10031), *Acinetobacter baumannii* DSM 30007, ATCC 19606, *Pseudomonas aeruginosa* CCM 3955 (ATCC 27853). The cultivation was done in Cation-adjusted Mueller-Hinton broth (CAMHB, M-H 2 Broth, Sigma-Aldrich) at 35 ± 2 °C. Tested compounds were dissolved in DMSO (Sigma-Aldrich, USA) to produce stock solutions. The final concentration of DMSO in the cultivation medium did not exceed 1% (v/v) of the total solution composition and did not affect the growth of bacteria. Positive controls consisted of test microbe solely, while negative controls consisted of cultivation medium and DMSO. Antibacterial activity was expressed as minimum inhibitory concentration (MIC, in μM) after 24 and 48 h of static incubation in the dark and humidified atmosphere at 35 ± 2 °C. Visual inspection and metabolic activity indicator, Alamar Blue (AlamarBlue^{TM} Cell Viability reagent, ThermoFisher Scientific, USA), were used for MIC endpoint evaluation. The internal quality standards gentamycin and ciprofloxacin (both from Sigma-Aldrich) were involved in assays (for MIC of standards, see TableS2). All experiments were conducted in duplicates. For the results to be valid, the difference in MIC for one compound determined from two parallel measurements must not be greater than one step on the dilution scale.

Table S2 *Results of internal quality controls (standards) in antibacterial screening.*

	**MINIMUM INHIBITORY CONCENTRATION**			
	ciprofloxacin (µg/mL)		gentamicin (µg/mL)	
**BACTERIAL STRAIN**	spectrophotometric detection*	visual detection**	spectrophotometric detection*	visual detection**
Staphylococcus aureus **subsp. *aureus* ATCC 29213, CCM 4223**	0.256	0.256	1	0.5
Staphylococcus aureus **subsp. *aureus,* MRSA, ATCC 43300, CCM 4750**	0.128	0.128	>8	>8
Staphylococcus epidermidis **ATCC 12228, CCM 4418**	0.256	0.128	0.125	0.125
***Enterococcus faecalis*, ATCC 29212, CCM 4224**	0.512	1.024	>8	>8
***Escherichia coli*, ATCC 25922, CCM 3954**	0.008	0.008	1	1
***Klebsiella pneumoniae*, ATCC 10031, CCM 4415**	0.008	0.008	0.5	0.5
***Acinetobacter baumannii*, ATCC 19606, DSM 30007**	0.512	0.256	8	8
***Pseudomonas aeruginosa*, ATCC 27853, CCM 3955**	0.512	0.512	0.5	0.5

*The MIC of antibacterial agents is the lowest concentration giving rise to an inhibition of growth of 95% of that of the drug-free control. Results were read after 24 h microdilution plates cultivation without agitation at 35 ± 2 °C in a humidified atmosphere. Measured on a microplate reader (Synergy^{TM} HTX, BioTek Instruments, Inc., USA) at wavelength 530 nm.

**The MIC was determined by naked eye in the well with the lowest drug concentration, where no visible growth of microbial agent was detected. Results were read after 24 h (bacteria, yeasts) or 48 h (moulds) microdilution plates cultivation without agitation at 35 ± 2 °C in humidified atmosphere.

1.1.4. *In vitro* antifungal evaluation

Antifungal activity evaluation was performed using a microdilution broth method according to EUCAST instructions (The European Committee on Antimicrobial Susceptibility Testing) with slight modifications.[2, 3] Eight fungal strains (four yeast and four mould strains) were used for antifungal activity screening, namely: *Candida albicans* CCM 8320 (ATCC 24433), *Candida krusei* CCM 8271 (ATCC 6258), *Candida parapsilosis* CCM 8260 (ATCC 22019), *Candida tropicalis* CCM 8264 (ATCC 750), *Aspergillus fumigatus* ATCC 204305, *Aspergillus flavus* CCM 8363, *Lichtheimia corymbifera* CCM 8077, and *Trichophyton interdigitale* CCM 8377 (ATCC 9533). Tested strains were purchased from the Czech Collection of Microorganisms (CCM, Brno, Czech Republic) or from the American Type Collection Cultures (ATCC, Manassas, VA, USA). Tested compounds were dissolved in DMSO and diluted in a twofold manner with RPMI 1640 medium, with glutamine and 2% glucose, buffered to pH 7.0 with MOPS (3-morpholinopropane-1-sulfonic acid). The final concentration of DMSO in the testing

medium did not exceed 1% (v/v) of the total solution composition. Static incubation was performed in the dark and in humid atmosphere, at 35 ± 2 °C, for 24 and 48 h (72 and 120 h for *Trichophyton interdigitale*). Positive controls consisted of test microbe solely, while negative controls consisted of cultivation medium and DMSO. Visual inspection and metabolic activity indicator, Alamar Blue (ThermoFisher Scientific), were used for MIC endpoint evaluation. The internal quality standards, amphotericin B (Sigma-Aldrich) and voriconazole (Toronto research Chemicals, CA) were involved in assays (for MIC of standards, see Table S3). All experiments were conducted in duplicates. For the results to be valid, the difference in MIC for one compound determined from two parallel measurements must not be greater than one step on the dilution scale.

Table S3 *Results of internal quality controls (standards) in antifungal screening*

	**MINIMUM INHIBITORY CONCENTRATION**			
	amphotericin B (µg/mL)		voriconazole (µg/mL)	
**YEAST/MOULD STRAIN**	spectrophoto-metric detection*	visual detection**	spectrophoto-metric detection***	visual detection**
***Candida albicans* ATCC 24433, CCM 8320**	0.5	0.5	0.03	>16
***Candida krusei* ATCC 6258, CCM 8271**	0.5	0.5	0.25	>16
***Candida parapsilosis* ATCC 22019, CCM 8260**	0.5	0.5	0.03	>16
***Candida tropicalis* ATCC 750, CCM 8264**	1	1	0.0625	>16
***Aspergillus fumigatus* ATCC 204305**	1	1	0.25	1
***Aspergillus flavus* CCM 8363**	4	4	1	>16
***Lichtheimia corymbifera* CCM 8077**	0.125	0.125	>16	>16
***Trichophyton interdigitale* ATCC 9533, CCM 8377**	1	1	>16	>16

*The MIC of amphotericin B is the lowest concentration giving rise to an inhibition of growth of 90% of that of the drug-free control. Results were read after 24 h (yeasts) or 48 h (moulds) microdilution plates cultivation without agitation at 35 ± 2 °C in humidified atmosphere. Measured on a microplate reader (Synergy^{TM} HTX, BioTek Instruments, Inc., USA) at wavelength 530 nm.

**The MIC was determined by naked eye in the well with the lowest drug concentration, where no visible growth of microbial agent was detected. Results were read after 24 h (bacteria, yeasts) or 48 h (moulds) of microdilution plates cultivation without agitation at 35 ± 2 °C in humidified atmosphere.

***The MIC of azole (voriconazole) antifungal agents is the lowest drug concentration giving inhibition of growth of 50% of that of the drug-free control. Results were read after 24 h (yeasts) or 48 h (moulds) microdilution plates cultivation without agitation at 35 ± 2 °C in humidified atmosphere. Measured on a microplate reader (Synergy^{TM} HTX, BioTek Instruments, Inc., USA) at wavelength 530 nm.

1.2. *In vitro* HepG2 cytotoxicity determination

The human hepatocellular liver carcinoma cell line HepG2 purchased from Health Protection Agency Culture Collections (ECACC, Salisbury, UK) was cultured in MEM (Minimum Essentials Eagle Medium) (Sigma-Aldrich) supplemented with 10% foetal bovine serum (PAA Laboratories GmbH, Pasching, Austria), 1% L-glutamine solution (Sigma-Aldrich), and non-essential amino acid solution (Sigma-Aldrich) in a humidified atmosphere containing 5% CO_2 at 37 °C. For subculturing, the cells were harvested after trypsin/EDTA (Sigma-Aldrich) treatment at 37 °C. To evaluate cytotoxicity, the cells treated with the tested substances were used as experimental groups, whereas untreated HepG2 cells served as controls.

The cells were seeded in a density of 10,000 cells per well in a 96-well plate. During the next day, the cells were treated with each of the tested substances dissolved in DMSO. The tested substances were prepared at different incubation concentrations (1–1000 µM) in triplicates according to their solubility. Simultaneously, the controls representing 100% cell viability, 0% cell viability (the cells treated with 10% DMSO), no cell control, and vehiculum controls were also prepared in triplicates. After 24 h of incubation in a humidified atmosphere containing 5% CO_2 at 37 °C, the reagent from the kit CellTiter 96 AQueous One Solution Cell Proliferation Assay (CellTiter 96; PROMEGA, Fitchburg, WI, USA) was added. After 2 h of incubation at 37 °C, the absorbance of the samples was recorded at 490 nm (TECAN, Infinita M200, Austria). A standard toxicological parameter IC_{50} was calculated by nonlinear regression from a semilogarithmic plot of incubation concentration versus the percentage of absorbance relative to untreated controls using GraphPad Prism 8 software (GraphPad Software, San Diego, CA, USA). The compounds were tested in two separate experiments in cell line passage 3 and 23.

2. Additional results

2.1. Analytical data

Abbreviations used in the text:

PzH	Hydrogen from pyrazine
ArH	Hydrogen from benzene ring
cHexH	Hydrogen from cyclohexane
PtH	Hydrogen from pteridine

Yields refer to isolated, chromatographically pure compounds and to the last single step of the synthesis.

3-benzamidopyrazine-2-carboxamide (1). White solid. Yield: 95%. mp 225.7–227.8 °C. ^{1}H NMR (500 MHz, DMSO-d_6) δ 12.82 (s, 1H, NHCO), 8.67 (d, *J* = 2.4 Hz, 1H, PzH), 8.63 (s, 1H, CONH), 8.42 (d, *J* = 2.4 Hz, 1H, PzH), 8.18 (s, 1H, CONH), 8.02–7.90 (m, 2H, ArH), 7.70–7.63 (m, 1H, ArH), 7.62–7.55 (m, 2H). ^{13}C NMR (126 MHz, DMSO-d_6) δ 168.32, 163.73, 148.97, 146.23, 137.91, 134.49, 132.68, 131.41, 129.19, 127.52, 39.20. IR (ATR-Ge, cm^{-1}): 3462, 3191, 3069, 1708 (C=O, CONH), 1697 (C=O, CONH), 1594, 1506, 1460, 1067. Anal. Calcd. For $C_{12}H_{10}N_4O_2$ (MW 242.24): C, 59.5; H, 4.16; N, 23.13. Found: C, 59.19; H, 3.91; N, 23.21.

3-(2-methylbenzamido)pyrazine-2-carboxamide (2). Beige solid. Yield: 95%. mp 234.4–235.9 °C. ^{1}H NMR (500 MHz, DMSO-d_6) δ 12.80 (s, 1H, NHCO), 8.66 (d, *J* = 2.4 Hz, 1H, PzH), 8.63 (s, 1H, CONH), 8.42 (d, *J* = 2.4 Hz, 1H, PzH), 8.18 (s, 1H, CONH), 7.80–7.73 (m, 2H, ArH), 7.52–7.43 (m, 2H, ArH), 2.41 (s, 3H, CH_3). ^{13}C NMR (126 MHz, DMSO-d_6) δ 168.35, 163.75, 148.99, 146.23, 138.55, 137.83, 134.49, 133.24, 131.27, 129.05, 128.04, 124.61, 21.16. IR (ATR-Ge, cm^{-1}): 3460, 3193, 1706 (C=O, CONH), 1674 (C=O, CONH), 1577, 1488, 1454, 1065. Anal. Calcd. For $C_{13}H_{12}N_4O_2$ (MW 256.27): C, 60.93; H, 4.72; N, 21.86. Found: C, 61.26; H, 4.75; N, 21.54.

3-(3-methylbenzamido)pyrazine-2-carboxamide (3). Beige solid. Yield: 96%. mp 226.9–228.8 °C. ^{1}H NMR (500 MHz, DMSO-d_6) δ 12.78 (s, 1H, NHCO), 8.66 (d, *J* = 2.4 Hz, 1H, PzH), 8.63 (s, 1H, CONH), 8.41 (d, *J* = 2.3 Hz, 1H, PzH), 8.17 (s, 1H, CONH), 7.87 (d, 2H, ArH), 7.39 (d, *J* = 7.9 Hz, 2H, ArH), 2.39 (s, 3H, CH_3). ^{13}C NMR (126 MHz, DMSO-d_6) δ 168.36, 163.57, 149.06, 146.24, 142.90, 137.76, 131.71, 131.21, 129.71, 127.56, 21.24. IR (ATR-Ge, cm^{-1}): 3463, 3246, 3078, 1692 (C=O, CONH), 1688 (C=O, CONH), 1588, 1481, 1458, 1078. Anal. Calcd. For $C_{13}H_{12}N_4O_2$ (MW 256.27): C, 60.93; H, 4.72; N, 21.86. Found: C, 61.21; H, 4.72; N, 22.06.

3-(4-methylbenzamido)pyrazine-2-carboxamide (4). Beige solid. Yield: 93%. mp 238.0–240.0 °C. ^{1}H NMR (500 MHz, DMSO-d_6) δ 12.78 (s, 1H, NHCO), 8.66 (d, *J* = 2.4 Hz, 1H, PzH), 8.63 (s, 1H, CONH), 8.41 (d, *J* = 2.3 Hz, 1H, PzH), 8.17 (s, 1H, CONH), 7.87 (d, 2H, ArH), 7.39 (d, *J* = 7.9 Hz, 2H, ArH), 2.39 (s, 3H, CH_3). ^{13}C NMR

(126 MHz, DMSO-d_6) δ 168.36, 163.57, 149.06, 146.24, 142.90, 137.76, 131.71, 131.21, 129.71, 127.56, 21.24. IR (ATR-Ge, cm^{-1}): 3460, 3181, 3035, 1695 (C=O, CONH), 1673 (C=O, CONH), 1591, 1495, 1459, 1080. Anal. Calcd. For $C_{13}H_{12}N_4O_2$ (MW 256.27): C, 60.93; H, 4.72; N, 21.86. Found: C, 61.12; H, 4.67; N, 21.95.

3-(4-ethylbenzamido)pyrazine-2-carboxamide (5). White solid. Yield: 95%. mp 215.5–218.5 °C. ^{1}H NMR (500 MHz, DMSO-d_6) δ 12.80 (s, 1H, NHCO), 8.67 (d, *J* = 2.3, 1H, PzH), 8.64 (s, 1H, CONH), 8.44 (d, *J* = 2.4Hz, 1H, PzH), 8.18 (s, 1H, CONH), 7.93–7.86 (m, 2H, ArH), 7.45–7.40 (m, 2H, ArH), 2.70 (q, *J* = 7.6 Hz, 2H, CH_2), 1.21 (td, *J* = 7.5, 0.5 Hz, 3H, CH_3). ^{13}C NMR (126 MHz, DMSO-d_6) δ 168.37, 163.61, 149.07, 148.99, 146.26, 137.78, 131.99, 131.22, 128.56, 127.67, 40.20, 28.28, 15.46. IR (ATR-Ge, cm^{-1}): 3463, 3190, 3079, 2973, 2930, 1695 (C=O, CONH), 1670 (C=O, CONH), 1596, 1495, 1458, 1072. Anal. Calcd. For $C_{14}H_{14}N_4O_2$ (MW 270.29): C, 62.21; H, 5.22; N, 20.73. Found: C, 61.98; H, 5.27; N, 19.92.

3-(4-(*tert*-butyl)benzamido)pyrazine-2-carboxamide (6). White solid. Yield: 90%. mp 210.0–211.5 °C. ^{1}H NMR (500 MHz, DMSO-d_6) δ 12.79 (s, 1H, NHCO), 8.66 (d, *J* = 2.4 Hz, 1H, PzH), 8.62 (s, 1H, CONH), 8.41 (d, *J* = 2.4 Hz, 1H, PzH), 17 (s, 1H, CONH), 7.97–7.88 (m, 2H, ArH), 7.68–7.55 (m, 2H, ArH), 1.31 (s, 9H, CH_3). ^{13}C NMR (126 MHz, DMSO-d_6) δ 168.31, 163.54, 155.63, 149.05, 146.23, 137.73, 131.75, 131.14, 127.40, 125.96, 34.94, 31.02. IR (ATR-Ge, cm^{-1}): 3461, 3202, 2966, 1703 (C=O, CONH), 1669 (C=O, CONH), 1585, 1486, 1458, 1072. Anal. Calcd. For $C_{16}H_{18}N_4O_2$ (MW 298.35): C, 64.41; H, 6.08; N, 18.78. Found: C, 64.22; H, 5.97; N, 18.82.

3-(2-methoxybenzamido)pyrazine-2-carboxamide (7). White solid. Yield: 98%. mp 205.4–206.7 °C. ^{1}H NMR (500 MHz, DMSO-d_6) δ 12.64 (s, 1H, NHCO), 8.63 (d, *J* = 2.4 Hz, 1H, Pz), 8.47 (s, 1H, CONH), 8.39 (d, *J* = 2.4 Hz, 1H, PzH), 8.04 (s, 1H. CONH), 7.91 (d, 1H, ArH), 7.57 (t, 1H, ArH), 7.22 (d, 1H, ArH), 7.10 (t, 1H, ArH), 3.98 (s, 3H, OCH_3). ^{13}C NMR (126 MHz, DMSO-d_6) δ 167.65, 162.74, 157.31, 148.42, 145.78, 137.66, 133.86, 131.73, 131.49, 122.48, 120.95, 112.41, 55.93. IR (ATR-Ge, cm^{-1}): 3458, 3201, 1703 (C=O, CONH), 1684 (C=O, CONH), 1599, 1475, 1451, 1288, 1087, 1021. Anal. Calcd. For $C_{13}H_{12}N_4O_3$ (MW 272.26): C, 57.35; H, 4.44; N, 20.58. Found: C, 57.14; H, 4.35; N, 20.3.

3-(3-methoxybenzamido)pyrazine-2-carboxamide (8). Beige solid. Yield: 95%. mp 221–223 °C. ^{1}H NMR (500 MHz, DMSO-d_6) δ 12.81 (s, 1H, NHCO), 8.67 (d, *J* = 2.4 Hz, 1H, PzH), 8.62 (s, 1H, CONH), 8.42 (d, *J* = 2.4 Hz, 1H, PzH), 8.18 (s, 1H, CONH), 7.57–7.47 (m, 3H, ArH), 7.22 (m, 1H, ArH), 3.84 (s, 3H, OCH_3). ^{13}C NMR (126 MHz, DMSO-d_6) δ 168.32, 163.42, 159.73, 148.88, 146.21, 137.91, 135.96, 131.43, 130.36, 119.57, 118.44, 112.70, 55.54. IR (ATR-Ge, cm^{-1}): 3455, 3218, 1702 (C=O, CONH), 1668 (C=O, CONH), 1588, 1486, 1460, 1266, 1065, 1035. Anal. Calcd. For $C_{13}H_{12}N_4O_3$ (MW 272.26): C, 57.35; H, 4.44; N, 20.58. Found: C, 57.04; H, 4.37; N, 20.11.

3-(4-methoxybenzamido)pyrazine-2-carboxamide (9). Beige solid. Yield: 94%. mp 257–258.5 °C. ^{1}H NMR (500 MHz, DMSO-d_6) δ 12.72 (s, 1H, CONH), 8.65 (d, *J* = 2.4 Hz, 1H, PzH), 8.62 (s, 1H, CONH), 8.40 (d, *J* = 2.4 Hz, 1H, PzH), 8.16 (s, 1H, CONH), 7.97–7.90 (m, 2H, ArH), 7.15–7.09 (m, 2H, ArH), 3.85 (s, 3H, OCH_3). ^{13}C NMR

(151 MHz, DMSO-d_6) δ 167.96, 162.80, 162.41, 148.80, 145.78, 137.14, 130.76, 129.10, 126.38, 114.04, 55.34. IR (ATR-Ge, cm^{-1}): 3459, 3155, 3048, 2964, 1695 (C=O, CONH), 1672 (C=O, CONH), 1607, 1490, 1248, 1025. Anal. Calcd. For $C_{13}H_{12}N_4O_3$ (MW 272.26): C, 57.35; H, 4.44; N, 20.58. Found: C, 57.42; H, 4.39; N, 20.17.

3-(2-fluorobenzamido)pyrazine-2-carboxamide (10). White solid. Yield: 90%. mp 211–213 °C. ^{1}H NMR (500 MHz, DMSO-d_6) δ 12.50 (s, 1H, CONH), 8.64 (d, J = 2.4 Hz, 1H, PzH), 8.56 (s, 1H, CONH), 8.44 (d, J = 2.4 Hz, 1H, PzH), 8.11 (s, 1H, CONH), 7.86 (t, 1H, ArH), 7.65 (m, 1H, ArH), 7.42–7.35 (m, 2H, ArH). ^{13}C NMR (126 MHz, DMSO-d_6) δ 167.88, 161.10, 158.59, 148.33, 146.02, 138.28, 134.26, 131.51, 130.83, 125.23, 123.24, 116.85. IR (ATR-Ge, cm^{-1}): 3467, 3133, 1703 (C=O, CONH), 1694 (C=O, CONH), 1612, 1494, 1483, 1071. Anal. Calcd. For $C_{12}H_9FN_4O_2$ (MW 260.23): C, 55.39; H, 3.49; N, 21.53. Found: C, 55.40; H, 3.38; N, 21.35.

3-(3-fluorobenzamido)pyrazine-2-carboxamide (11). Beige solid. Yield: 95%. mp 234.1–236 °C. ^{1}H NMR (500 MHz, DMSO-d_6) δ 12.82 (s, 1H, CONH), 8.67 (s, 1H, PzH), 8.63 (s, 1H, CONH), 8.45 (s, 1H, PzH), 8.17 (s, 1H, CONH), 7.81 (d, 1H, ArH), 7.75–7.61 (m, 2H, ArH), 7.51 (t, 1H, ArH). ^{13}C NMR (151 MHz, DMSO-d_6) δ 168.45, 161.73, 148.73, 146.44, 138.84, 138.45, 135.41, 132.86, 132.11, 127.30, 126.70. IR (ATR-Ge, cm^{-1}): 3453, 3201,3078, 1713 (C=O, CONH), 1677 (C=O, CONH), 1589, 1506, 1486, 1063. Anal. Calcd. For $C_{12}H_9FN_4O_2$ (MW 260.23): C, 55.39; H, 3.49; N, 21.53. Found: C, 55.36; H, 3.43; N, 21.17.

3-(4-fluorobenzamido)pyrazine-2-carboxamide (12). Beige solid. Yield: 93%. mp 209–211 °C. ^{1}H NMR (600 MHz, DMSO-d_6) δ 12.74 (s, 1H, CONH), 8.63 (d, J = 2.5 Hz, 1H, PzH), 8.58 (s, 1H, CONH), 8.39 (d, J = 2.5 Hz, 1H, PzH), 8.13 (s, 1H, CONH), 8.06–8.01 (m,2H, ArH), 7.46–7.40 (m, 2H, ArH). ^{13}C NMR (151 MHz, DMSO-d_6) δ 168.63, 165.91, 163.09, 149.19, 146.54, 138.35, 132.03, 131.38, 130.71, 116.62. IR (ATR-Ge, cm^{-1}): 3451, 3203, 1707 (C=O, CONH), 1695 (C=O, CONH), 1601, 1498, 1495, 1069. Anal. Calcd. For $C_{12}H_9FN_4O_2$ (MW 260.23): C, 55.39; H, 3.49; N, 21.53. Found: C, 55.47; H, 3.34; N, 21.59.

3-(2-chlorobenzamido)pyrazine-2-carboxamide (13). White solid. Yield: 96%. mp 238–240 °C. ^{1}H NMR (500 MHz, DMSO-d_6) δ 12.26 (s, 1H, NHCO), 8.56 (d, J = 2.4 Hz, 2H, PzH, CONH), 8.43 (d, J = 2.4 Hz, 1H, PzH), 8.11 (s, 1H, CONH), 7.66 (dd, 1H, ArH), 7.59–7.51 (m, 2H, ArH), 7.48 (td, 1H, ArH). ^{13}C NMR (151 MHz, DMSO-d_6) δ 165.63, 164.77, 145.32, 144.88, 140.29, 138.39, 134.97, 131.82, 130.13, 129.93, 129.22, 127.28. IR (ATR-Ge, cm^{-1}): 3465, 3077, 1715 (C=O, CONH), 1676 (C=O, CONH), 1573, 1497, 1455, 1070. Anal. Calcd. For $C_{12}H_9ClN_4O_2$ (MW 276.68): C, 52.09; H, 3.28; N, 20.25. Found: C, 51.81; H, 3.19; N, 19.95.

3-(3-chlorobenzamido)pyrazine-2-carboxamide (14). Beige solid. Yield: 96%. mp 256.7–258 °C. ^{1}H NMR (500 MHz, DMSO-d_6) δ 12.81 (s, 1H, NHCO), 8.67 (s, 1H, PzH), 8.62 (s, 1H, CONH), 8.45 (s, 1H, PzH), 8.17 (s, 1H, CONH), 7.94 (d, 2H, ArH), 7.68 (d, 2H, ArH). ^{13}C NMR (126 MHz, DMSO-d_6) δ 168.20, 162.30, 148.57, 146.13, 138.20, 136.68, 135.29, 131.97, 131.38, 130.25, 126.54, 122.34. IR (ATR-Ge, cm^{-1}): 3454, 3219, 3036, 1713

(C=O, CONH), 1698 (C=O, CONH), 1589, 1498, 1479, 1063. Anal. Calcd. For $C_{12}H_9ClN_4O_2$ (MW 276.68): C, 52.09; H, 3.28; N, 20.25. Found: C, 51.67; H, 3.13; N, 20.05.

3-(4-chlorobenzamido)pyrazine-2-carboxamide (15). White crystals. Yield: 95%. mp 238–240 °C. ^{1}H NMR (500 MHz, DMSO-d_6) δ 12.83 (s, 1H, NHCO), 8.67 (d, J = 2.3 Hz, 1H, PzH), 8.65 (s, 1H, CONH), 8.44 (d, J = 2.4 Hz, 1H, PzH), 8.19 (s, 1H, CONH), 8.00–7.94 (m, 2H, ArH), 7.71–7.63 (m, 2H, ArH). ^{13}C NMR (126 MHz, DMSO-d_6) δ 168.08, 162.64, 148.57, 146.02, 137.96, 137.35, 133.08, 131.62, 129.29, 129.13. IR (ATR-Ge, cm^{-1}): 3451, 3205, 3050, 1696 (C=O, CONH), 1671 (C=O, CONH), 1595, 1489, 1459, 1070. Anal. Calcd. For $C_{12}H_9ClN_4O_2$ (MW 276.68): C, 52.09; H, 3.28; N, 20.25. Found: C, 51.89; H, 3.41; N, 19.87.

3-(2-bromobenzamido)pyrazine-2-carboxamide (16). White solid. Yield: 98%. mp 223.1–225.1 °C. 1H NMR (500 MHz, DMSO-d_6) δ 12.22 (s, 1H, NHCO), 8.56 (d, J = 2.6 Hz, 2H, PzH, CONH), 8.43 (d, J = 2.4 Hz, 1H, PzH), 8.11 (s, 1H, CONH), 7.72 (dd, J = 7.9, 1.2 Hz, 1H, ArH), 7.63 (dd, 1H, ArH), 7.52 (m, 1H, ArH), 7.45 (m, 1H, ArH). ^{13}C NMR (126 MHz, DMSO-d_6) δ 167.87, 165.04, 147.98, 145.95, 138.43, 138.31, 133.32, 132.01, 131.71, 129.03, 128.18, 118.80. IR (ATR-Ge, cm^{-1}): 3466, 3200, 1715 (C=O, CONH), 1673 (C=O, CONH), 1589, 1490, 1455, 1070. Anal. Calcd. For $C_{12}H_9BrN_4O_2$ (MW 321.13): C, 44.88; H, 2.82; N, 17.45. Found: C, 45.25; H, 3.00; N, 16.35.

3-(3-bromobenzamido)pyrazine-2-carboxamide (17). White solid. Yield: 98%. mp 255.0–256.0°C. ^{1}H NMR (500 MHz, DMSO-d_6) δ 12.82 (s, 1H, NHCO), 8.67 (d, J = 2.5 Hz, 1H, PzH), 8.62 (s, 1H, CONH), 8.44 (d, J = 2.4 Hz, 1H, PzH), 8.19 (s, 1H, CONH), 8.10 (s, 1H, ArH), 7.95 (d, 1H, ArH), 7.86 (d, 1H, ArH), 7.56 (t, 1H, ArH). ^{13}C NMR (126 MHz, DMSO-d_6) δ 168.22, 162.32, 148.59, 146.15, 138.22, 136.69, 135.31, 131.99, 131.40, 130.26, 126.56, 122.36. IR (ATR-Ge, cm^{-1}): 3469, 3455, 3222, 3065, 1712 (C=O, CONH), 1674 (C=O, CONH), 1575, 1498, 1477, 1069. Anal. Calcd. For $C_{12}H_9BrN_4O_2$ (MW 321.13): C, 44.88; H, 2.82; N, 17.45. Found: C, 45.13; H, 2.64; N, 17.27.

3-(4-bromobenzamido)pyrazine-2-carboxamide (18). Beige solid. Yield: 99%. mp 254.5–255.6 °C. 1H NMR (600 MHz, DMSO-d_6) δ 12.77 (s, 1H, NHCO), 8.63 (d, J = 2.1 Hz, 1H, PzH), 8.58 (s, 1H, CONH), 8.40 (d, J = 2.1 Hz, 1H, PzH), 8.13 (s, 1H, CONH), 7.86 (m, 2H, ArH), 7.78 (m, 2H, ArH). 13C NMR (151 MHz, DMSO-d6) δ 168.60, 163.31, 149.09, 146.54, 138.47, 133.98, 132.59, 132.12, 129.94, 126.87. ^{13}C NMR (151 MHz, DMSO-d_6) δ 168.60, 163.31, 149.09, 146.54, 138.47, 133.98, 132.59, 132.12, 129.94, 126.87. IR (ATR-Ge, cm^{-1}): 3452, 3197, 3035, 1709 (C=O, CONH), 1670 (C=O, CONH), 1590, 1482, 1460, 1071. Anal. Calcd. For $C_{12}H_9BrN_4O_2$ (MW 321.13): C, 44.88; H, 2.82; N, 17.45. Found: C, 45.27; H, 2.76; N, 17.32.

3-(3,5-dichlorobenzamido)pyrazine-2-carboxamide (19). White solid. Yield: 96%. mp >300 °C. ^{1}H NMR (500 MHz, DMSO-d_6) δ 12.79 (s, 1H, NHCO), 8.68 (d, J = 2.4 Hz, 1H, PzH), 8.62 (s, 1H, CONH), 8.47 (d, J = 2.5 Hz, 1H, PzH), 8.19 (s, 1H, CONH), 8.09–7.82 (m, 3H, ArH). ^{13}C NMR (151 MHz, DMSO-d_6) δ 167.69, 160.94, 147.92,

145.68, 138.10, 137.59, 134.61, 132.14, 131.40, 125.95. IR (ATR-Ge, cm^{-1}): 3453, 3261, 3082, 1714 (C=O, CONH), 1677 (C=O, CONH), 1569, 1495, 1458, 1066. Anal. Calcd. For $C_{12}H_8Cl_2N_4O_2$ (MW 311.12): C, 46.33; H, 2.59; N, 18.01. Found: C, 46.07; H, 2.3; N, 17.91.

3-(2-(trifluoromethyl)benzamido)pyrazine-2-carboxamide (20). Beige solid. Yield: 96%. mp 201.5–202.4 °C. ^{1}H NMR (600 MHz, DMSO-d_6) δ 12.24 (s, 1H, NHCO), 8.55 (d, J = 2.5 Hz, 2H, PzH, CONH), 8.43 (d, J = 2.5 Hz, 1H, PzH), 8.10 (s, 1H, CONH), 7.87 (d, 1H, ArH), 7.84–7.78 (m, 1H, ArH), 7.77 (d, 1H, ArH), 7.74 (t, 1H, ArH). ^{13}C NMR (151 MHz, DMSO-d_6) δ 168.20, 165.43, 148.15, 146.21, 138.91, 136.20, 133.38, 132.53, 131.12, 128.58, 126.30, 125.12. IR (ATR-Ge, cm^{-1}): 3478, 3195, 1716 (C=O, CONH), 1674 (C=O, CONH), 1587, 1486, 1456, 1072. Anal. Calcd. For $C_{13}H_9F_3N_4O_2$ (MW 310.24): C, 50.33; H, 2.92; N, 18.06. Found: C, 50.19; H, 2.83; N, 18.64.

3-(4-nitrobenzamido)pyrazine-2-carboxamide (21). Orange solid. Yield: 95%. mp >300 °C. ^{1}H NMR (600 MHz, DMSO-d_6) δ 12.88 (s, 1H, NHCO), 8.65 (d, J = 2.4 Hz, 1H, PzH), 8.60 (s, 1H, CONH), 8.44 (d, J = 2.4 Hz, 1H, PzH), 8.42–8.34 (m, 2H, ArH), 8.18–8.12 (m, 3H, ArH, CONH). ^{13}C NMR (151 MHz, DMSO-d_6) δ 168.48, 162.78, 150.18, 148.78, 146.51, 140.36, 138.88, 132.65, 129.44, 124.69. IR (ATR-Ge, cm^{-1}): 3408, 3205, 1698 (C=O, CONH), 1674 (C=O, CONH), 1602, 1516, 1487, 1323, 1081. Anal. Calcd. For $C_{12}H_9N_5O_4$ (MW 287.24): C, 50.18; H, 3.16; N, 24.38. Found: C, 51.25; H, 3.15; N, 23.69.

3-(2-hydroxybenzamido)pyrazine-2-carboxamide (22). White solid. Yield: 18%. mp 243.7 – 245.1°C. ^{1}H NMR (500 MHz, DMSO-D_6) δ 12.72 (s, 1H, NHCO), 11.54 (s, 1H, OH), 8.66 (d, J = 2.4 Hz, 1H, PzH), 8.50 (s, 1H, CONH), 8.43 (d, J = 2.4 Hz, 1H, PzH), 8.03 (s, 1H, CONH), 7.87 (dd, J = 7.9, 1.7 Hz, 1H, ArH), 7.49–7.42 (m, 1H, ArH), 7.04–6.95 (m, 2H, ArH). ^{13}C NMR (126 MHz, DMSO-D_6) δ 167.62, 165.16, 158.31, 148.13, 145.81, 138.09, 134.33, 132.71, 129.75, 119.53, 118.27, 117.48, 40.19. IR (ATR-Ge, cm^{-1}): IR_not measured. Anal. Calcd. For $C_{12}H_{10}N_4O_3$ (MW 258.24): C, 55.81; H, 3.9; N, 21.7. Found: C, 55.66; H, 3.68; N, 21.58.

3-(4-hydroxybenzamido)pyrazine-2-carboxamide (23). Beige solid. Yield: 11%. mp >300 °C. ^{1}H NMR (500 MHz, DMSO-D_6) δ 12.67 (s, 1H, NHCO), 10.31 (s, 1H, OH), 8.64 (d, J = 2.4 Hz, 1H, PzH), 8.62 (s, CONH), 8.38 (d, J = 2.3 Hz, 1H, PzH), 8.16 (s, 1H, CONH), 7.88–7.81 (m, 2H, ArH), 6.96–6.89 (m, 2H, ArH). ^{13}C NMR (126 MHz, DMSO-D_6) δ 168.25, 163.13, 161.37, 149.10, 146.07, 137.28, 130.80, 129.55, 124.85, 115.54. IR (ATR-Ge, cm^{-1}): IR_not measured. Anal. Calcd. For $C_{12}H_{10}N_4O_3$ (MW 258.24): C, 55.81; H, 3.9; N, 21.7. Found: C, 55.81; H, 3.74; N, 21.32.

3-(4-aminobenzamido)pyrazine-2-carboxamide (24). Yellow solid. Yield: 6%. mp >300 °C. ^{1}H NMR (600 MHz, DMSO-D_6) δ 12.53 (s, 1H, NHCO), 8.61 (d, J = 2.4 Hz, 1H, PzH), 8.59 (s, 1H, CONH), 8.34 (d, J = 2.4 Hz, 1H, PzH), 8.13 (s, 1H, CONH), 7.70–7.67 (m, 2H, ArH), 6.66–6.63 (m, 2H, ArH), 5.95 (s, 2H, NH_2). ^{13}C NMR (151 MHz, DMSO-D_6) δ 168.34, 163.25, 153.00, 149.42, 146.11, 130.28, 129.23, 129.09, 120.35, 112.92. IR (ATR-Ge, cm^{-1}):

IR_not measured. Anal. Calcd. For $C_{12}H_{11}N_5O_2$ (MW 257.25): C, 56.03; H, 4.31; N, 27.22. Found: C, 57.52; H, 4.31; N, 27.22.

3-(2-(4-fluorophenyl)acetamido)pyrazine-2-carboxamide (25). White solid. Yield: 40%. mp 225.3–227.3 °C. ^{1}H NMR (500 MHz, DMSO-*d₆*) δ 11.76 (s, 1H, NHCO), 8.59 (d, *J* = 2.4 Hz, 1H, PzH), 8.47 (s, 1H, CONH), 8.37 (d, *J* = 2.4 Hz, 1H, PzH), 8.06 (s, 1H, CONH), 7.40–7.32 (m, 2H, ArH), 7.19–7.11 (m, 2H, ArH), 3.93 (s, 2H, CH_2). ^{13}C NMR (151 MHz, DMSO-d_6) δ 169.08, 167.44, 161.86, 160.25, 147.83, 145.33, 137.22, 131.22, 130.90, 114.87, 42.98. IR (ATR-Ge, cm^{-1}): 3458, 3195, 3037, 1704 (C=O, CONH), 1674 (C=O, CONH), 1579, 1506. Anal. Calcd. For $C_{13}H_{11}FN_4O_2$ (274.26): C, 56.93; H, 4.04; N, 20.43. Found: C, 57.23; H, 4.12; N, 20.32.

3-(3-phenylpropanamido)pyrazine-2-carboxamide (26). Beige solid Yield: 93%. mp 160.3–161.5 °C. ^{1}H NMR (500 MHz, DMSO-d_6) δ 11.72 (s, 1H, NHCO), 8.56 (d, *J* = 2.4 Hz, 1H, PzH), 8.48 (m, 1H, CONH), 8.35 (d, *J* = 2.4 Hz, 1H), 8.06 (s, 1H, CONH), 7.33–7.23 (m, 4H, ArH), 7.26–7.13 (m, 1H, ArH), 3.01–2.87 (m, 4H, 2 X CH_2). ^{13}C NMR (126 MHz, DMSO-d_6) δ 171.09, 167.77, 147.97, 145.54, 140.98, 137.20, 130.92, 128.31, 125.94, 32.21, 30.18. IR (ATR-Ge, cm^{-1}): 3465, 3197, 3084, 1716 (C=O, CONH), 1674 (C=O, CONH), 1602, 1544, 1506,1072. Anal. Calcd. For $C_{14}H_{14}FN_4O_2$ (270.29) C, 62.21; H, 5.22; N, 20.73. Found: C, 62.32; H, 4.96; N, 20.88.

3-(cyclohexanecarboxamido)pyrazine-2-carboxamide (27). Beige solid. Yield: 98%. mp 105.5–107.0 °C. ^{1}H NMR (600 MHz, DMSO-d_6) δ 11.74 (s, 1H, NHCO), 8.57 (d, *J* = 2.4 Hz, 1H, PzH), 8.47 (s, 1H, CONH), 8.35 (d, *J* = 2.3 Hz, 1H, PzH), 8.03 (s, 1H, CONH), 2.56–2.49 (m, 1H, cHexH), 1.93–1.85 (m, 2H, cHexH), 1.78–1.69 (m, 2H, cHexH), 1.67–1.59 (m, 1H, cHexH), 1.45–1.35 (m, 2H, cHexH), 1.34–1.24 (m, 2H, cHexH), 1.24–1.13 (m 1H, cHexH). ^{13}C NMR (151 MHz, DMSO-d_6) δ 173.49, 167.83, 148.25, 145.70, 137.23, 131.23, 55.81, 45.40, 28.77, 25.07. IR (ATR-Ge, cm^{-1}): 3465, 3204, 3080, 2929, 2854, 1708 (C=O, CONH), 1675 (C=O, CONH), 1602, 1506. Anal. Calcd. For $C_{12}H_{16}N_4O_2$ (MW 248.29): C, 58.05; H, 6.5; N, 22.57. Found: C, 57.71; H, 6.41; N, 22.34.

3-(adamantane-1-carboxamido)pyrazine-2-carboxamide (28). Beige solid. Yield:90%. mp 212.6–214.1 °C. ^{1}H NMR (600 MHz, DMSO-d_6) δ 12.10 (s, 1H, NHCO), 8.58 (d, *J* = 2.5 Hz, 1H, PzH), 8.56 (s, 1H, CONH), 8.34 (d, *J* = 2.5 Hz, 1H, PzH), 8.10 (s, 1H, CONH), 2.07–2.02 (m, 3H), 1.92–1.88 (m, 6H), 1.77–1.65 (m, 6H). ^{13}C NMR (151 MHz, DMSO-d_6) δ 175.14, 168.64, 149.59, 146.52, 137.67, 130.85, 42.38, 39.02, 36.50, 28.09. IR (ATR-Ge, cm^{-1}): 3457, 3225, 2904, 2850, 1683 (C=O, CONH), 1585 (C=O, CONH), 1489, 1453. Anal. Calcd. For $C_{16}H_{20}N_4O_2$ (MW 300.36): C, 63.98; H, 6.71; N, 18.65. Found: C, 63.40; H, 6.63; N, 18.42.

3-butyramidopyrazine-2-carboxamide (29). White solid. Yield: 85%. mp 158.7–159.5 °C. ^{1}H NMR (600 MHz, DMSO-d_6) δ 11.65 (s, 1H, NHCO), 8.57 (d, *J* = 2.4 Hz, 1H, PzH), 8.46 (s, 1H, CONH), 8.35 (d, *J* = 2.4 Hz, 1H, PzH), 8.03 (s, 1H, CONH), 2.56 (t, *J* = 7.4 Hz, 2H, CH_2), 1.62 (h, *J* = 7.4 Hz, 2H, CH_2), 0.92 (t, *J* = 7.4 Hz, 3H, CH_3). ^{13}C NMR (151 MHz, DMSO-d_6) δ 171.47, 167.79, 148.03, 145.56, 137.17, 131.05, 17.84, 13.55. IR (ATR-Ge, cm^{-1}):

3419, 3205, 2972, 2880, 1667 (C=O, CONH), 1581 (C=O, CONH), 1489. Anal. Calcd. For $C_9H_{12}N_4O_2$ (MW 208.22): C, 51.92; H, 5.81; N, 26.91. Found: C, 52.36; H, 5.72; N, 27.37.

3-hexanamidopyrazine-2-carboxamide (30). White crystals. Yield: 90%. mp 138.4–139.0 °C. ^{1}H NMR (500 MHz, DMSO-*d₆*) δ 11.66 (s, 1H, NHCO), 8.57 (d, *J* = 2.4 Hz, 1H, PzH), 8.46 (s, 1H, CONH), 8.35 (d, *J* = 2.4 Hz, 1H, PzH), 8.04 (s, 1H, CONH), 2.57 (t, 2H, CH_2), 1.64–1.55 (m, 2H, CH_2), 1.35–1.24 (m, 4H, 2x CH_2), 0.91–0.82 (m, 3H, CH_3). ^{13}C NMR (126 MHz, DMSO-d_6) δ 171.75, 167.97, 148.22, 145.74, 137.34, 131.20, 37.48, 30.94, 24.26, 22.05, 13.98. IR (ATR-Ge, cm^{-1}): 3426, 3201, 2966, 2939, 2872, 1665 (C=O, CONH), 1578 (C=O, CONH), 1506. Anal. Calcd. For $C_{11}H_{16}N_4O_2$ (MW 236.28): C, 55.92; H, 6.83; N, 23.71. Found: C, 55.92; H, 6.87; N, 23.74

3-heptanamidopyrazine-2-carboxamide (31). White solid. Yield: 92%. mp 131.5–132.1 °C. ^{1}H NMR (600 MHz, DMSO-d_6) δ 11.66 (s, 1H, NHCO), 8.56 (d, *J* = 2.4 Hz, 1H, PzH), 8.46 (s, 1H, CONH), 8.35 (d, *J* = 2.4 Hz, 1H, PzH), 8.04 (s, 1H, CONH), 2.57 (t, 2H, CH_2), 1.64–1.54 (m, 2H, CH_2), 1.35–1.19 (m, 6H, 3x CH_2), 0.89–0.81 (m, 3H, CH_3). ^{13}C NMR (151 MHz, DMSO-d_6) δ 171.57, 167.79, 148.05, 145.57, 137.16, 130.99, 37.35, 31.12, 28.51, 28.45, 24.41, 22.03, 13.91. IR (ATR-Ge, cm^{-1}): 3406, 3206, 2956, 2918, 2852, 1668 (C=O, CONH), 1579 (C=O, CONH), 1487. Anal. Calcd. For $C_{12}H_{18}N_4O_2$ (MW 250.3): C, 57.58; H, 7.25; N, 22.38. Found: C, 58.8; H, 7.62; N, 21.4.

3-octanamidopyrazine-2-carboxamide (32). Beige solid. Yield: 90%. mp 127.9–129.3 °C. ^{1}H NMR (600 MHz, DMSO-d_6) δ 11.65 (s, 1H, NHCO), 8.57 (d, *J* = 2.5 Hz, 1H, PzH), 8.46 (s, 1H, CONH), 8.35 (d, *J* = 2.5 Hz, 1H, PzH), 8.03 (s, 1H, CONH), 2.57 (t, *J* = 7.4 Hz, 2H, CH_2), 1.59 (p, *J* = 7.4 Hz, 2H, CH_2), 1.39–1.21 (m, 8H, 4x CH_2), 0.88–0.83 (m, 3H, CH_3). ^{13}C NMR (151 MHz, DMSO-d_6) δ 171.59, 167.80, 148.04, 145.58, 143.69, 137.19, 131.06, 37.36, 31.02, 28.21, 24.37, 21.96, 13.90. IR (ATR-Ge, cm^{-1}): 3429, 3201, 2959, 2921, 2855, 1680 (C=O, CONH), 1665 (C=O, CONH), 1580, 1507. Anal. Calcd. For $C_{13}H_{20}N_4O_2$ (MW 264.33): C, 59.07; H, 7.63; N, 21.2. Found: C, 57.38; H, 7.18; N, 21.96.

N-methyl-3-(4-methylbenzamido)pyrazine-2-carboxamide (33). Yellow solid. Yield: 89%. mp 178.4–179.5°C. ^{1}H NMR (600 MHz, DMSO-d_6) δ 12.80 (s, 1H, NHCO), 9.26 (q, *J* = 4.9 Hz, 1H, CONH), 8.66 (d, *J* = 2.4 Hz, 1H, PzH), 8.41 (d, *J* = 2.4 Hz, 1H, PzH), 7.90–7.85 (m, 2H, ArH), 7.43–7.38 (m, 2H, ArH), 2.85 (d, *J* = 4.8 Hz, 3H, CH_3), 2.40 (s, 3H). ^{13}C NMR (151 MHz, DMSO-d_6) δ 165.89, 163.33, 148.55, 145.90, 142.70, 137.46, 131.50, 130.89, 129.54, 127.32, 26.15, 21.05. IR (ATR-Ge, cm^{-1}): 3406, 2926, 1693 (C=O, CONH), 1660 (C=O, CONH), 1593, 1548, 1496, 1449, 1073. Anal. Calcd. For $C_{14}H_{14}N_4O_2$ (MW 270.29): C, 62.21; H, 5.22; N, 20.73. Found: C, 62.30; H, 5.27; N, 20.07.

3-(4-chlorobenzamido)-*N*-methylpyrazine-2-carboxamide (34). Beige solid. Yield: 85%. mp 204.2–205.1 °C. ^{1}H NMR (500 MHz, DMSO-d_6) δ 12.83 (s, 1H, NHCO), 9.27 (q, *J* = 4.8 Hz, 1H, CONH), 8.66 (d, *J* = 2.4 Hz, 1H, PzH), 8.43 (d, *J* = 2.4 Hz, 1H, PzH), 8.02–7.94 (m, 2H, ArH), 7.71–7.65 (m, 2H, ArH), 2.84 (d, *J* = 4.8 Hz, 3H, CH_3). ^{13}C NMR (126 MHz, DMSO-d_6) δ 166.25, 162.98, 148.71, 146.32, 138.26, 137.81, 133.47, 131.79, 129.65, 129.59,

39.45, 26.63. IR (ATR-Ge, cm^{-1}): 3399, 3355, 3155, 1709 (C=O, CONH), 1674 (C=O, CONH), 1591, 1486, 1451, 1071. Anal. Calcd. For $C_{13}H_{11}ClN_4O_2$ (MW 290.71): C, 53.71; H, 3.81; N, 19.27. Found: C, 53.47; H, 3.62; N, 19.30.

3-(4-bromobenzamido)-*N*-methylpyrazine-2-carboxamide (35). Beige solid. Yield: 92%. mp 202.0–202.5 °C. ^{1}H NMR (600 MHz, DMSO-d_6) δ 12.79 (s, 1H, NHCO), 9.22 (q, *J* = 4.8 Hz, 1H, CONH), 8.63 (d, *J* = 2.4 Hz, 1H, PzH), 8.39 (d, *J* = 2.4 Hz, 1H, PzH), 7.89–7.84 (m, 2H, ArH), 7.81–7.76 (m, 2H, ArH), 2.80 (d, *J* = 4.8 Hz, 3H, CH_3). ^{13}C NMR (151 MHz, DMSO-d_6) δ 166.34, 163.22, 148.79, 146.41, 138.35, 133.94, 132.62, 131.89, 129.88, 126.89, 26.71. IR (ATR-Ge, cm^{-1}): 3356, 3062, 1707 (C=O, CONH), 1647 (C=O, CONH), 1588, 1481, 1450, 1075. Anal. Calcd. For $C_{13}H_{11}BrN_4O_2$ (MW 335.16): C, 46.59; H, 3.31; N, 16.72. Found: C, 46.52; H, 3.32; N, 15.61.

N,*N*-dimethyl-3-(4-methylbenzamido)pyrazine-2-carboxamide (36). Beige solid. Yield: 91%. mp 188.7–189.5 °C. ^{1}H NMR (600 MHz, DMSO-d_6) δ 11.06 (s, 1H, NHCO), 8.58 (d, *J* = 2.6 Hz, 1H, PzH), 8.48 (d, *J* = 2.5 Hz, 1H, PzH), 7.89 (d, *J* = 7.9 Hz, 2H, ArH), 7.33 (d, *J* = 7.9 Hz, 2H, ArH), 3.05 (s, 3H, CH_3), 2.91 (s, 3H, CH_3), 2.39 (s, 3H, CH_3). ^{13}C NMR (151 MHz, DMSO-d_6) δ 167.23, 166.48, 146.28, 144.42, 143.56, 143.04, 139.55, 130.85, 129.50, 128.74, 38.91, 35.08, 21.60. IR (ATR-Ge, cm^{-1}): 3270, 2932, 1672 (C=O, CONH), 1629 (C=O, CONH), 1529, 1487, 1397, 1062. Anal. Calcd. For $C_{15}H_{16}N_4O_2$ (MW 284.32): C, 63.37; H, 5.67; N, 19.71. Found: C, 63.16; H, 5.59; N, 19.63.

3-(4-chlorobenzamido)-*N*,*N*-dimethylpyrazine-2-carboxamide (37). White solid. Yield: 92%. mp 184.1–185.3 °C. ^{1}H NMR (600 MHz, DMSO-d_6) δ 11.21 (s, 1H, CONH), 8.55 (d, *J* = 2.5 Hz, 1H, PzH), 8.46 (d, *J* = 2.5 Hz, 1H, PzH), 7.97–7.92 (m, 2H, ArH), 7.59–7.54 (m, 2H, ArH), 3.00 (s, 3H, CH_3), 2.87 (s, 3H, CH_3). ^{13}C NMR (151 MHz, DMSO-d_6) δ 167.14, 165.73, 146.02, 144.46, 143.66, 139.85, 137.76, 132.43, 130.60, 129.12, 38.90, 35.09. IR (ATR-Ge, cm^{-1}): 3234, 1673 (C=O, CONH), 1630 (C=O, CONH), 1597, 1533, 1487, 1448, 1093. Anal. Calcd. For $C_{14}H_{13}ClN_4O_2$ (MW 304.73): C, 55.18; H, 4.30; N, 18.39. Found: C, 54.84; H, 4.16; N, 18.02.

3-(cyclohexanecarboxamido)-*N*-(2,3-dihydro-1*H*-inden-2-yl)pyrazine-2-carboxamide (38). White solid. Yield: 93%. mp 148.5–151.0 °C. ^{1}H NMR (500 MHz, CDCl3) δ 11.98 (s, 1H, NHCO), 8.58 (d, *J* = 2.3 Hz, 1H, PzH), 8.33 (d, *J* = 8.1 Hz, 1H, CONH), 8.12 (d, *J* = 2.3 Hz, 1H, PzH), 7.29–7.27 (m, 2H, indane ArH), 7.24–7.20 (m, 2H, indane ArH), 4.94 – 4.85 (m, 1H, indane CH), 3.44 (dd, *J* = 16.1, 7.2 Hz, 2H, indane CH2), 2.98 (dd, *J* = 16.1, 4.8 Hz, 2H, indane CH2), 2.48–2.41 (m, 1H, cHexH), 2.12–2.01 (m, 2H, cHexH), 1.91–1.80 (m, 2H, cHexH), 1.74–1.55 (m, 3H, cHexH), 1.42–1.23 (m, 3H, cHexH). 13C NMR (126 MHz, CDCl3) δ 174.53, 165.16, 149.59, 146.42, 140.42, 136.13, 129.01, 126.96, 124.81, 50.55, 47.36, 39.95, 29.34, 25.67, 25.61. Anal. Calcd. For $C_{21}H_{24}N_4O_2$ (MW 364.45): C, 69.21; H, 6.64; N, 15.37; O. Found: C, 69.10; H, 6.61; N, 15,06.

2-phenylpteridin-4(3*H*)-one (39). White solid. Yield: 95%. mp 283.5–284.5 °C. 1H NMR (500 MHz, DMSO-d_6) δ 13.07 (s, 1H, PtH), 9.01 (d, *J* = 2.1 Hz, 1H, PtH), 8.82 (d, *J* = 2.1 Hz, 1H, PtH), 8.25–8.20 (m, 2H, ArH), 7.72–

7.49 (m, 3H, ArH). ^{13}C NMR (126 MHz, DMSO-d_6) δ 161.54, 155.88, 155.03, 150.45, 144.11, 133.15, 132.24, 132.03, 128.75, 128.29. IR (ATR-Ge, cm^{-1}): 3129, 1682 (C=O, CONH), 1601, 1543, 1478, 1465, 1038. Anal. Calcd. For $C_{12}H_8N_4O$ (MW 224.22): C, 64.28; H, 3.6; N, 24.99. Found: C, 63.14; H, 3.42; N, 24.14. HPLC purity 100%

2-(*p*-tolyl)pteridin-4(3*H*)-one (40). White solid. Yield: 95%. mp 300.5–302 °C. 1H NMR (500 MHz, DMSO-d_6) δ 12.97 (s, 1H, PtH), 8.99 (d, *J* = 2.1 Hz, 1H, PtH), 8.80 (d, *J* = 2.1 Hz, 1H, PtH), 8.14 (d, *J* = 8.0 Hz, 2H, ArH), 7.38 (d, *J* = 7.9 Hz, 2H, ArH), 2.40 (s, 3H, Me). ^{13}C NMR (126 MHz, DMSO-d_6) δ 161.49, 155.72, 155.09, 150.39, 143.89, 142.51, 133.03, 129.32, 129.15, 128.22, 21.05. IR (ATR-Ge, cm^{-1}): 3040, 1707 (C=O, CONH), 1611, 1600, 1563, 1297, 1037. Anal. Calcd. For $C_{13}H_{10}N_4O$ (MW 238.25): C, 65.54; H, 4.23; N, 23.52. Found: C, 63.25; H, 4.19; N, 22.63. HPLC purity 100%

2-(4-fluorophenyl)pteridin-4(3*H*)-one (41). White solid. Yield: 95%. mp 277.0–279.5 °C. 1H NMR (500 MHz, DMSO-d_6) δ 13.08 (s, 1H, PtH), 9.00 (d, *J* = 2.0 Hz, 1H, PtH), 8.82 (d, *J* = 2.1 Hz, 1H, PtH), 8.34–8.26 (m, 2H, ArH), 7.48–7.39 (m, 2H, ArH). ^{13}C NMR (126 MHz, DMSO-d_6) δ 165.25, 163.25, 161.09, 154.65, 150.08, 143.78, 132.74, 130.75, 130.68, 128.29, 128.27, 115.55, 115.38. IR (ATR-Ge, cm^{-1}): 3120, 1685 (C=O, CONH), 1613, 1580, 1544, 1519,1479, 1456, 1034. Anal. Calcd. For $C_{12}H_7FN_4O$ (MW 242.21): C, 59.51; H, 2.91; N, 23.13. Found: C, 57.83; H, 2.92; N, 22.53. HPLC purity 100%

2-(4-chlorophenyl)pteridin-4(3*H*)-one (42). White solid. Yield: 95%: mp 306.5–307.9 °C. 1H NMR (500 MHz, DMSO-d_6) δ 13.04 (s, 1H, PtH), 9.00 (d, *J* = 2.1 Hz, 1H, PtH), 8.82 (d, *J* = 2.1 Hz, 1H, PtH), 8.27–8.21 (m, 2H, ArH), 7.68–7.62 (m, 2H, ArH). ^{13}C NMR (126 MHz, DMSO-d_6) δ 161.22, 154.82, 150.26, 144.06, 137.05, 133.03, 130.78, 129.98, 128.66. IR (ATR-Ge, cm^{-1}): 3119, 1686 (C=O, CONH), 1603, 1562, 1544, 1475, 1456, 1092. Anal. Calcd. For $C_{12}H_7ClN_4O$ (MW 258.67): C, 55.72; H, 2.73; N, 21.66. Found: C, 52.11; H, 2.89; N, 20.25. HPLC purity 100%

2-(4-bromophenyl)pteridin-4(3*H*)-one (43). White solid. Yield: 95%. mp 315.4–317.4 °C. 1H NMR (500 MHz, DMSO-d_6) δ 13.14 (s, 1H, PtH), 9.01 (d, *J* = 2.0 Hz, 1H, PtH), 8.83 (d, *J* = 2.0 Hz, 1H, PtH), 8.20–8.12 (m, 2H, ArH), 7.84–7.77 (m, 2H, ArH). ^{13}C NMR (126 MHz, DMSO-d_6) δ 161.27, 155.01, 154.79, 150.27, 144.08, 133.07, 131.62, 131.21, 130.16, 126.01. IR (ATR-Ge, cm^{-1}): 3119, 1684 (C=O, CONH), 1601, 1589, 1560, 1543, 1072. Anal. Calcd. For $C_{12}H_7BrN_4O$ (MW 303.12): C, 47.55; H, 2.33; N, 18.48. Found: C, 45.92; H, 2.44; N, 17.67. HPLC purity 100%

Methyl 3-benzamidopyrazine-2-carboxylate (1e). Beige solid. Yield: 64%. mp 133.8–136.1 °C. 1H NMR (500 MHz, DMSO-d6) δ 11.40 (s, 1H, NHCO), 8.72 (d, *J* = 2.4 Hz, 1H, PzH), 8.56 (d, *J* = 2.4 Hz, 1H, PzH), 8.05–7.99 (m, 2H, ArH), 7.68–7.61 (m, 1H, ArH), 7.59–7.52 (m, 2H, ArH), 3.76 (s, 3H, OCH_3). ^{13}C NMR (126 MHz, DMSO-d_6) δ 166.19, 164.93, 145.93, 145.25, 139.88, 138.49, 132.92, 132.57, 128.60, 128.09, 52.39, 39.61. IR

(ATR-Ge, cm^{-1}): 3312, 1687 (C=O, CONH), 1603, 1511, 1490, 1448, 1120. Anal. Calcd. For $C_{13}H_{11}N_3O_3$ (MW 257.25): C, 60.70; H, 4.31; N, 16.33. Found: C, 60.67; H, 4.21; N, 16.33.

Methyl 3-(4-methylbenzamido)pyrazine-2-carboxylate (4e). Beige solid. Yield: 80%. mp 158.9–161.2 °C. 1H NMR (500 MHz, DMSO-d6) δ 11.31 (s, 1H, NHCO), 8.71 (d, *J* = 2.4 Hz, 1H, PzH), 8.55 (d, *J* = 2.4 Hz, 1H, PzH), 7.96–7.90 (m, 2H, ArH), 7.38–7.32 (m, 2H, ArH), 3.75(s, 3H, OCH_3), 2.39 (s, 3H, CH_3). 13C NMR (126 MHz, DMSO-d_6) δ 166.00, 164.92, 146.02, 145.20, 142.82, 139.73, 138.44, 130.09, 129.12, 128.16, 52.35, 21.09. IR (ATR-Ge, cm^{-1}): 3313, 1687 (C=O, CONH), 1579, 1492, 1467, 1441, 1115. Anal. Calcd. For $C_{14}H_{13}N_3O_3$ (MW 271.28): C, 61.99; H, 4.83; N, 15.49. Found: C, 61.56; H, 4.67; N, 15.35.

Methyl 3-(4-methoxybenzamido)pyrazine-2-carboxylate (9e). White solid. Yield: 82%. mp 144.5–145.0 °C. 1H NMR (500 MHz, DMSO-d6) δ 11.23 (s, 1H, NHCO), 8.70 (d, *J* = 2.4 Hz, 1H, PzH), 8.53 (d, *J* = 2.4 Hz, 1H, PzH), 8.06–7.99 (m, 2H, ArH), 7.11–7.05 (m, 2H, ArH), 3.85 (s, 3H, OCH_3), 3.74 (s, 3H, OCH_3). 13C NMR (126 MHz, DMSO-d6) δ 165.46, 164.93, 162.68, 146.11, 145.10, 139.52, 138.38, 130.20, 124.90, 113.83, 55.51, 52.28. IR (ATR-Ge, cm^{-1}): 3307, 1704 (C=O, CONH), 1607, 1496, 1443, 1127. Anal. Calcd. For $C_{14}H_{13}N_3O_4$ (MW 287.28): C, 58.53; H, 4.56; N, 14.63. Found: C, 58.40; H, 4.42; N, 14.66.

Methyl 3-(2-fluorobenzamido)pyrazine-2-carboxylate (10e). Beige solid. Yield: 85%. mp 123.2–125.4 °C. 1H NMR (600 MHz, DMSO-d6) δ 11.42 (s, 1H, NHCO), 8.70 (d, *J* = 2.5 Hz, 1H, PzH), 8.57 (d, *J* = 2.5 Hz, 1H, PzH), 7.69 (t, 1H, ArH), 7.66–7.60 (m, 1H, ArH), 7.39–7.33 (m, 2H, ArH), 3.80 (s, 3H, OCH_3). ^{13}C NMR (151 MHz, DMSO-D_6) δ 165.00, 163.69, 160.32, 158.66, 145.42, 140.39, 140.27, 137.98, 133.80, 130.34, 124.84, 122.99, 116.51, 52.59. IR (ATR-Ge, cm^{-1}): 3254, 1712 (C=O, CONH), 1610, 1506, 1453, 1127. Calcd. For $C_{13}H_{10}FN_3O_3$ (MW 275.24): C, 56.73; H, 3.66; N, 15.27. Found: C, 57.1; H, 3.45; N, 15.19.

Methyl 3-(3-fluorobenzamido)pyrazine-2-carboxylate (11e). Beige Solid. Yield: 80%. mp 135.5–136.3 °C. ^{1}H NMR (600 MHz, DMSO-d_6) δ 11.48 (s, 1H, NHCO), 8.74 (d, *J* = 2.5 Hz, 1H PzH), 8.59 (d, *J* = 2.5 Hz, 1H, PzH), 7.87 (dt, *J* = 7.5, 1.3 Hz, 1H, ArH), 7.85–7.79 (m, 1H, ArH), 7.66–7.58 (m, 1H) ArH, 7.56–7.47 (m, 1H, ArH), 3.77 (s, 3H, OCH_3). ^{13}C NMR (151 MHz, DMSO-D_6) δ 164.83, 162.76, 161.14, 145.64, 145.34, 140.24, 138.63, 135.15, 130.83, 124.30, 119.58, 114.95, 52.48. IR (ATR-Ge, cm^{-1}): 3264, 1689, 1584, 1499, 1448, 1117. Anal. Calcd. For $C_{13}H_{10}FN_3O_3$ (MW 275.24): C, 56.73; H, 3.66; N, 15.27. Found: C, 56.71; H, 3.56; N, 15.30.

Methyl 3-(4-fluorobenzamido)pyrazine-2-carboxylate (12e). White solid. Yield: 85%. mp 151.1–153.6 °C. ^{1}H NMR (500 MHz, DMSO-d_6) δ 11.48 (s, 1H, NHCO), 8.73 (d, *J* = 2.4 Hz, 1H, PzH), 8.57 (d, *J* = 2.4 Hz, 1H, PzH), 7.98–7.91 (m, 2H, ArH), 7.80–7.74 (m, 2H, ArH), 3.75 (s, 3H, OCH_3). ^{13}C NMR (126 MHz, DMSO-d_6) δ 165.42, 164.82, 145.72, 145.26, 140.05, 138.55, 132.04, 131.65, 130.15, 126.47, 52.39. IR (ATR-Ge, cm^{-1}): 3271, 1689 (C=O, CONH), 1602, 1501, 1442, 1121. Anal. Calcd. For $C_{13}H_{10}FN_3O_3$ (MW 275.24): C, 56.73; H, 3.66; N, 15.27. Found: C, 56.55; H, 3.61; N, 15.29.

Methyl 3-(2-chlorobenzamido)pyrazine-2-carboxylate (13e). White solid. Yield: 84%. mp 114.5–115.6 °C. ^{1}H NMR (600 MHz, DMSO-*d$_6$*) δ 11.53 (s, 1H, NHCO), 8.70 (d, *J* = 2.2 Hz, 1H, PzH), 8.58 (d, *J* = 2.4 Hz, 1H, PzH), 7.60–7.53 (m, 3H, ArH), 7.51–7.47 (m, 1H, ArH), 3.82 (s, 3H, OCH_3). ^{13}C NMR (151 MHz, DMSO-*D$_6$*) δ 165.63, 164.77, 145.31, 144.89, 140.28, 138.39, 134.97, 131.82, 130.14, 129.93, 129.22, 127.28, 52.47. IR (ATR-Ge, cm^{-1}): 3330, 1702 (C=O, CONH), 1575, 1494, 1456, 1122. Anal. Calcd. For $C_{13}H_{10}ClN_3O_3$ (MW 291.69): C, 53.53; H, 3.46; N, 14.41. Found: C, 53.70; H, 3.28; N, 14.48.

Methyl 3-(3-chlorobenzamido)pyrazine-2-carboxylate (14e). White solid. Yield: 60%. mp 146.3–147.1 °C. ^{1}H NMR (500 MHz, DMSO-*d$_6$*) δ 11.53 (s, 1H, NHCO), 8.74 (d, *J* = 2.4 Hz, 1H, PzH), 8.59 (d, *J* = 2.4 Hz, 1H, PzH), 8.05 (t, 1H, ArH), 7.99–7.94 (m, 1H, ArH), 7.75–7.69 (m, 1H, ArH), 7.59 (t, 1H, ArH), 3.76 (s, 3H, OCH_3). ^{13}C NMR (126 MHz, DMSO-*d$_6$*) δ 165.13, 165.03, 145.79, 145.53, 140.41, 138.87, 135.05, 133.62, 132.61, 130.85, 128.06, 127.04, 52.67. IR (ATR-Ge, cm^{-1}): IR_not measured. Anal. Calcd. For $C_{13}H_{10}ClN_3O_3$ (MW 291.69): C, 53.53; H, 3.46; N, 14.41. Found: C, 53.49; H, 3.41; N, 14.76.

Methyl 3-(2-bromobenzamido)pyrazine-2-carboxylate (16e). White solid. Yield: 84%. mp 168.4–169.2 °C. ^{1}H NMR (500 MHz, DMSO-*d$_6$*) δ 11.53 (s, 1H, NHCO), 8.70 (d, *J* = 2.4 Hz, 1H, PzH), 8.58 (d, *J* = 2.4 Hz, 1H, PzH), 7.73 (d, 1H, ArH), 7.57–7.50 (m, 2H, ArH), 7.50–7.42 (m, 1H, ArH), 3.83 (s, 3H, OCH_3). ^{13}C NMR (126 MHz, DMSO-*d$_6$*) δ 166.42, 164.81, 145.33, 144.89, 140.30, 138.39, 137.08, 133.05, 131.91, 129.20, 127.76, 118.88, 52.53. IR (ATR-Ge, cm^{-1}): 3303, 1702 (C=O, CONH), 1574, 1492, 1469, 1455, 117. Anal. Calcd. For $C_{13}H_{10}BrN_3O_3$ (MW 336.15): C, 46.45; H, 3.00; N, 12.50. Found: C,46.93; H, 2.96; N, 12.19.

Methyl 3-(3-bromobenzamido)pyrazine-2-carboxylate (17e). White solid. Yield: 85%. mp 123.3–125.2 °C. 1H NMR (600 MHz, DMSO-d6) δ 11.50 (s, 1H, NHCO), 8.74 (d, *J* = 2.4 Hz, 1H, PzH), 8.59 (d, *J* = 2.3 Hz, 1H, PzH), 8.19 (t, 1H, ArH), 8.02–7.97 (m, 1H, ArH), 7.88–7.83 (m, 1H, ArH), 7.53 (t, 1H, ArH), 3.76 (s, 3H, OCH_3). ^{13}C NMR (151 MHz, DMSO-*D$_6$*) δ 164.81, 145.58, 145.31, 140.18, 138.66, 135.26, 135.04, 130.85, 130.69, 127.18, 121.81, 52.43. IR (ATR-Ge, cm^{-1}): 3302, 1717 (C=O, CONH), 1681, 1497 1452, 1124. Anal. Calcd. For $C_{13}H_{10}BrN_3O_3$ (MW 336.15): C, 46.45; H, 3.00; N, 12.50. Found: C, 46.97; H, 2.75; N, 1241.

Methyl 3-(4-bromobenzamido)pyrazine-2-carboxylate (18e). Beige Solid. Yield: 85%. mp 195.1–197.0 °C. ^{1}H NMR (500 MHz, DMSO-*d$_6$*) δ 11.48 (s, 1H, NHCO), 8.73 (d, *J* = 2.4 Hz, 1H, PzH), 8.57 (d, *J* = 2.4 Hz, 1H, PzH), 7.98–7.91 (m, 2H, ArH), 7.80–7.74 (m, 2H, ArH), 3.75 (s, 3H, OCH_3). ^{13}C NMR (126 MHz, DMSO-*d$_6$*) δ 165.42, 164.82, 145.72, 145.26, 140.05, 138.55, 132.04, 131.65, 130.15, 126.47, 52.39. IR (ATR-Ge, cm^{-1}): 3214, 1702 (C=O, CONH), 1594, 1508, 1483, 1454, 1128. Anal. Calcd. For $C_{13}H_{10}BrN_3O_3$ (MW 336.15): C, 46.45; H, 3.00; N, 12.50. Found: C, 47.15; H, 2.96; N, 12.32.

Methyl 3-(cyclohexanecarboxamido)pyrazine-2-carboxylate (27e). Beige solid. Yield: 82%. mp 106.9–108.3 °C. ^{1}H NMR (600 MHz, DMSO-*d$_6$*) δ 10.84 (s, 1H, NHCO), 8.62 (d, *J* = 2.2 Hz, 1H, PzH), 8.47 (d, *J* = 2.1 Hz, 1H, PzH),

3.74 (s, 3H, OCH_3), 2.50–2.43 (m, 1H, CH), 1.82–1.71 (m, 4H, 2xCH_2), 1.66–1.59 (m, 1H, CH), 1.41–1.31 (m, 2H, CH_2), 1.31–1.13 (m, 3H, CH_3). ^{13}C NMR (151 MHz, DMSO-D_6) δ 174.89, 164.71, 145.14, 144.88, 139.33, 138.23, 52.08, 43.71, 28.61, 25.30, 24.99. IR (ATR-Ge, cm^{-1}): 3314, 2932, 2858, 1719, (C=O, CONH) 1688, 1502, 1412, 1110. Anal. Calcd. For $C_{13}H_{17}N_3O_3$ (MW 263.3): C, 59.30; H, 6.51; N, 15.96. Found: C, 59.15; H, 6.42; N, 15.94.

Methyl 3-(adamantane-1-carboxamido)pyrazine-2-carboxylate (28e). Beige solid. Yield: 85%. mp 225.6–227.2 °C. ^{1}H NMR (600 MHz, DMSO-d_6) δ 10.42 (s, 1H, NHCO), 8.65 (d, *J* = 2.4 Hz, 1H, PzH), 8.48 (d, *J* = 2.4 Hz, 1H, PzH), 3.76 (s, 3H, OCH_3), 2.02 (q, *J* = 3.2 Hz, 3H, 3xCH), 1.90 (d, *J* = 3.2 Hz, 6H, 3xCH_2), 1.70 (t, *J* = 3.0 Hz, 6H, 3xCH_2). ^{13}C NMR (151 MHz, DMSO-D_6) δ 176.36, 164.81, 146.09, 145.02, 139.41, 138.06, 52.22, 40.74, 37.83, 35.90, 27.50. IR (ATR-Ge, cm^{-1}): 3318, 2905, 2891, 2819, 1717 (C=O, CONH), 1690, 1494, 1444, 1117. Anal. Calcd. For $C_{17}H_{21}N_3O_3$ (MW 315.37): C, 64.74; H, 6.71; N, 13.32. Found: C, 64.59; H, 6.66; N, 13.15.

2.2. Representative NMR spectra

Compound **4**

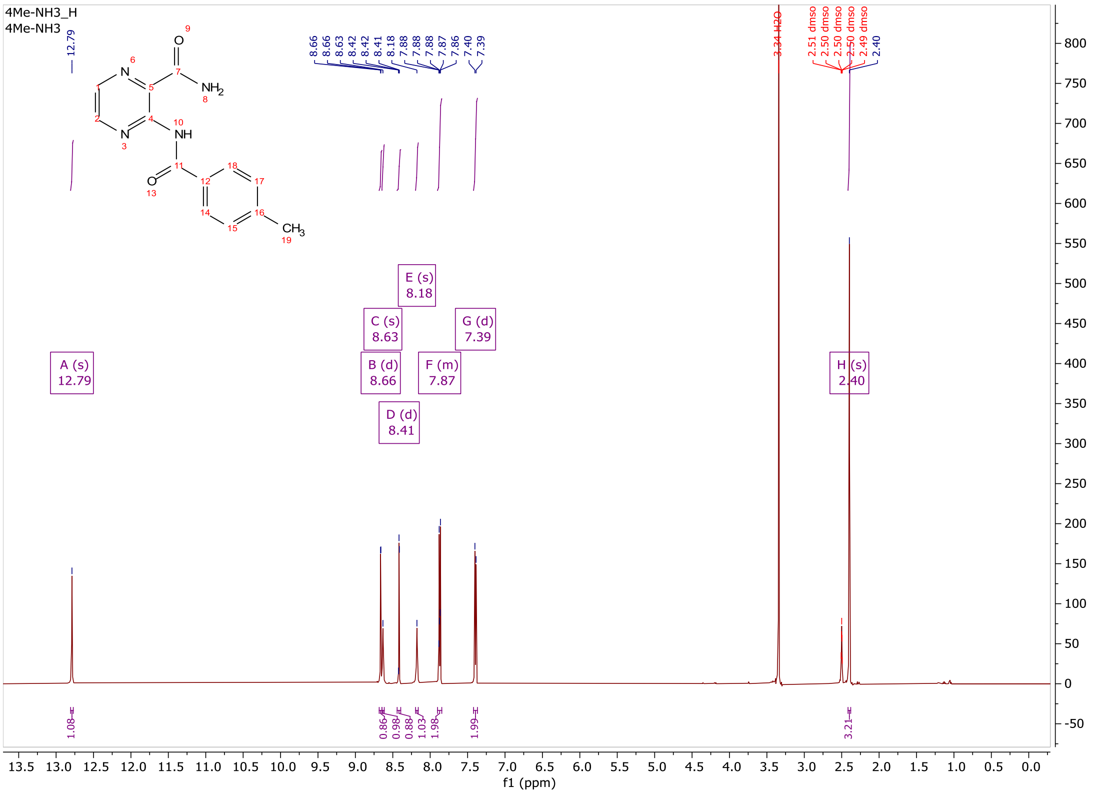

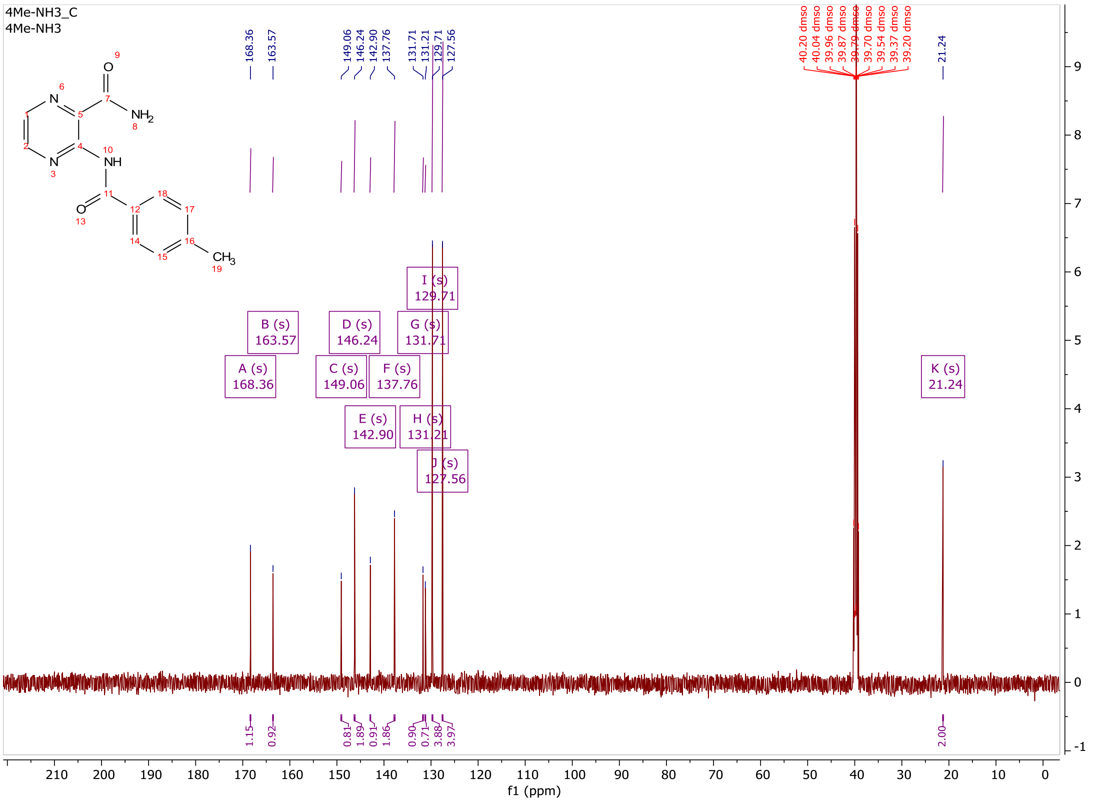

Compound **33**

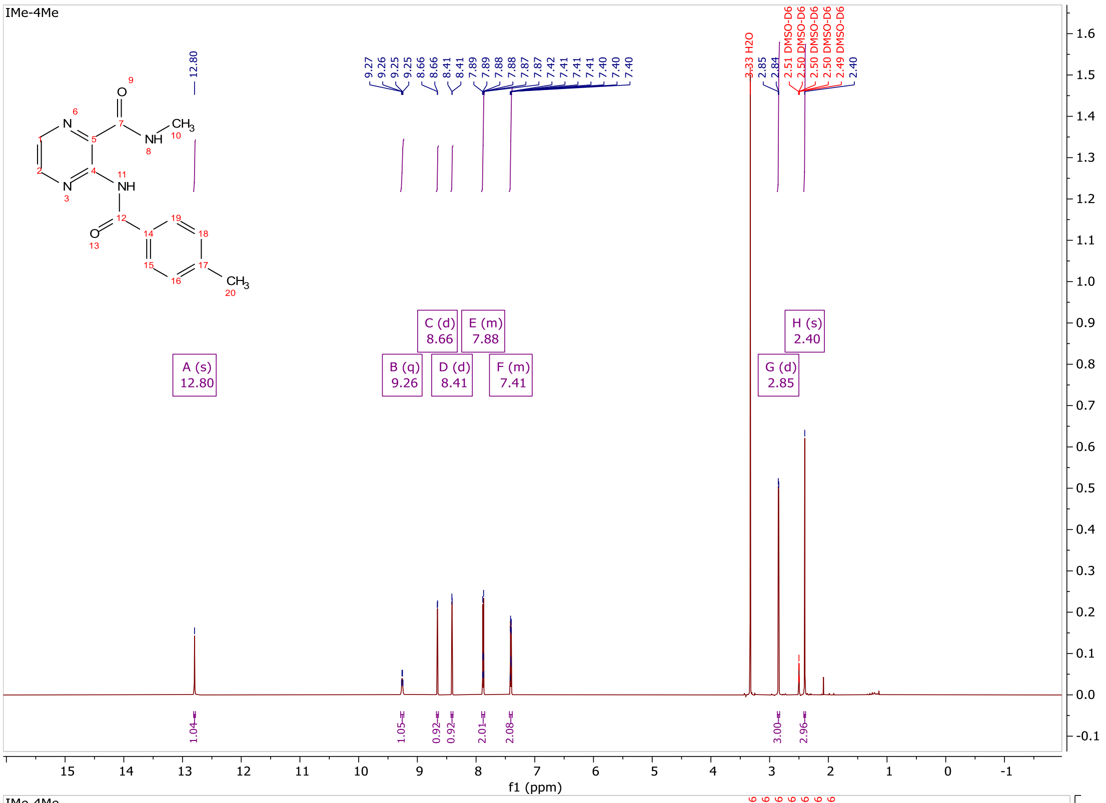

IMe-4Me
A (s) 12.80
B (q) 9.26
C (d) 8.66
D (d) 8.41
E (m) 7.88
F (m) 7.41
G (d) 2.85
H (s) 2.40
3.33 H2O
f1 (ppm)

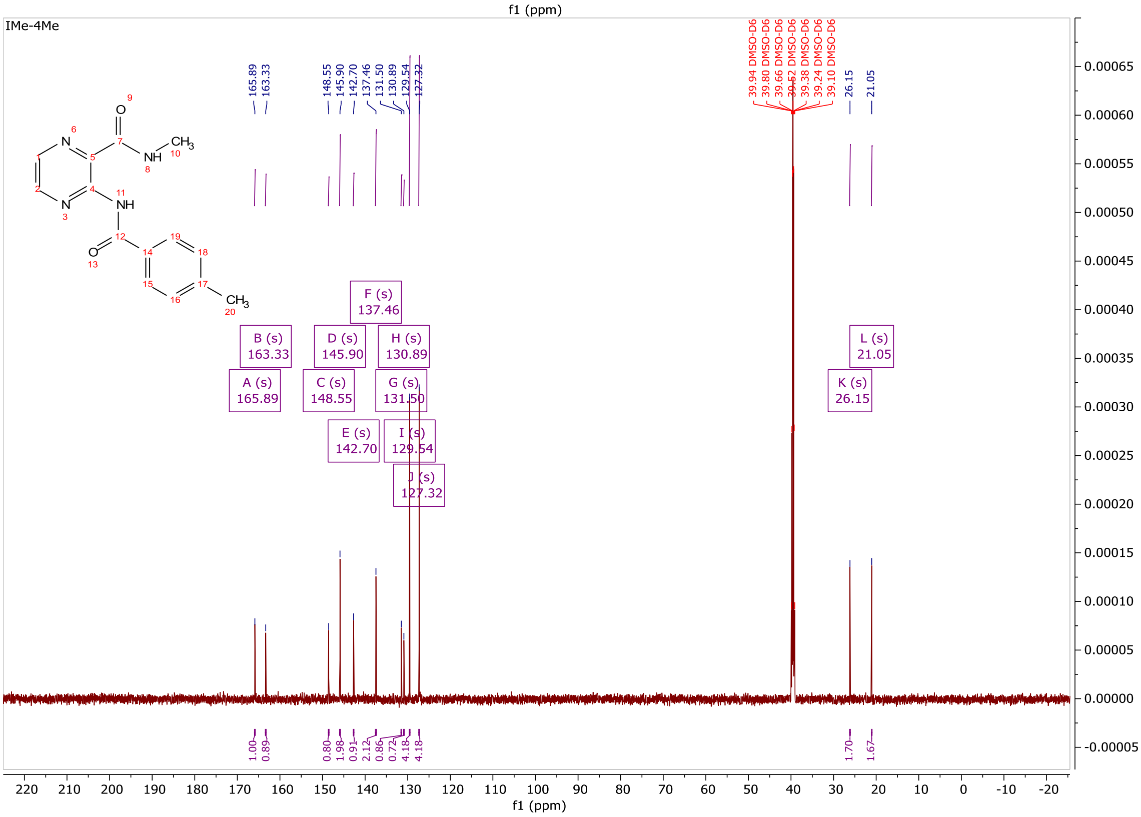

IMe-4Me
A (s) 165.89
B (s) 163.33
C (s) 148.55
D (s) 145.90
E (s) 142.70
F (s) 137.46
G (s) 131.50
H (s) 130.89
I (s) 129.54
J (s) 127.32
K (s) 26.15
L (s) 21.05
f1 (ppm)

Compound **36**

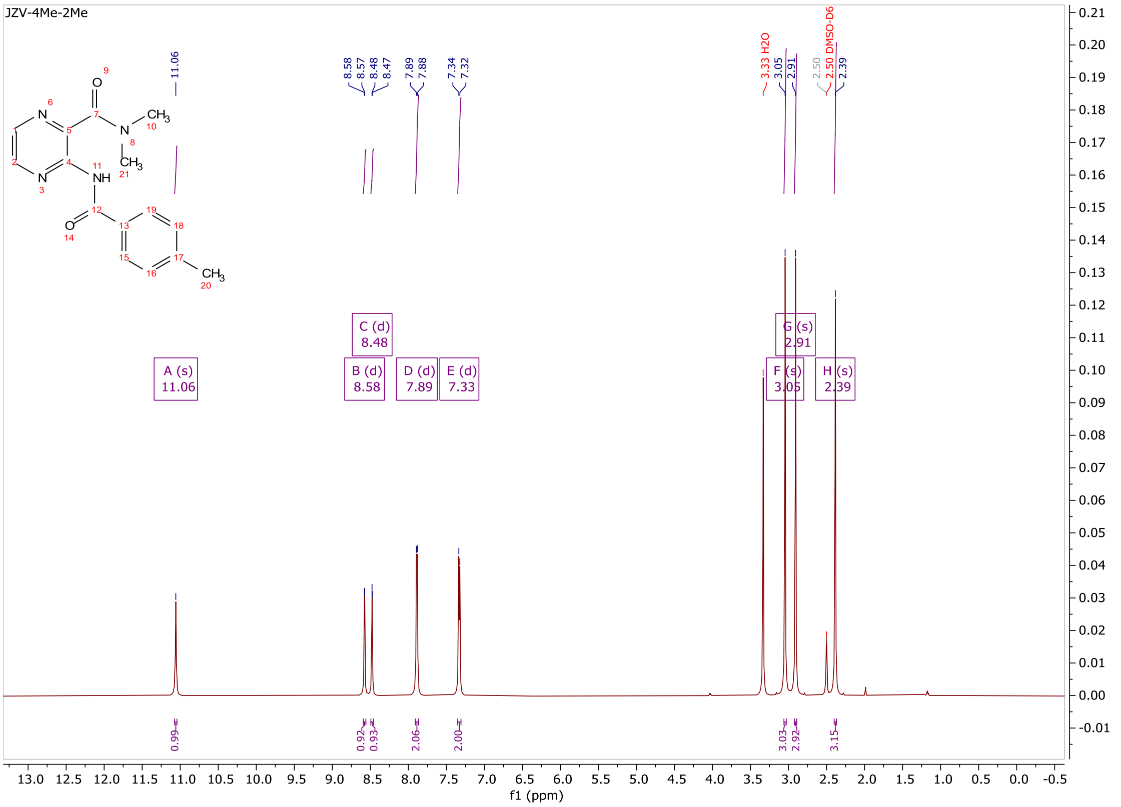

JZV-4Me-2Me
11.06
8.58
8.57
8.48
8.47
7.89
7.88
7.34
7.32
3.33 H2O
3.05
2.91
2.50
2.50 DMSO-D6
2.39
A (s)
11.06
B (d)
8.58
C (d)
8.48
D (d)
7.89
E (d)
7.33
F (s)
3.05
G (s)
2.91
H (s)
2.39
0.99
0.92
0.93
2.06
2.00
3.03
2.92
3.15
f1 (ppm)

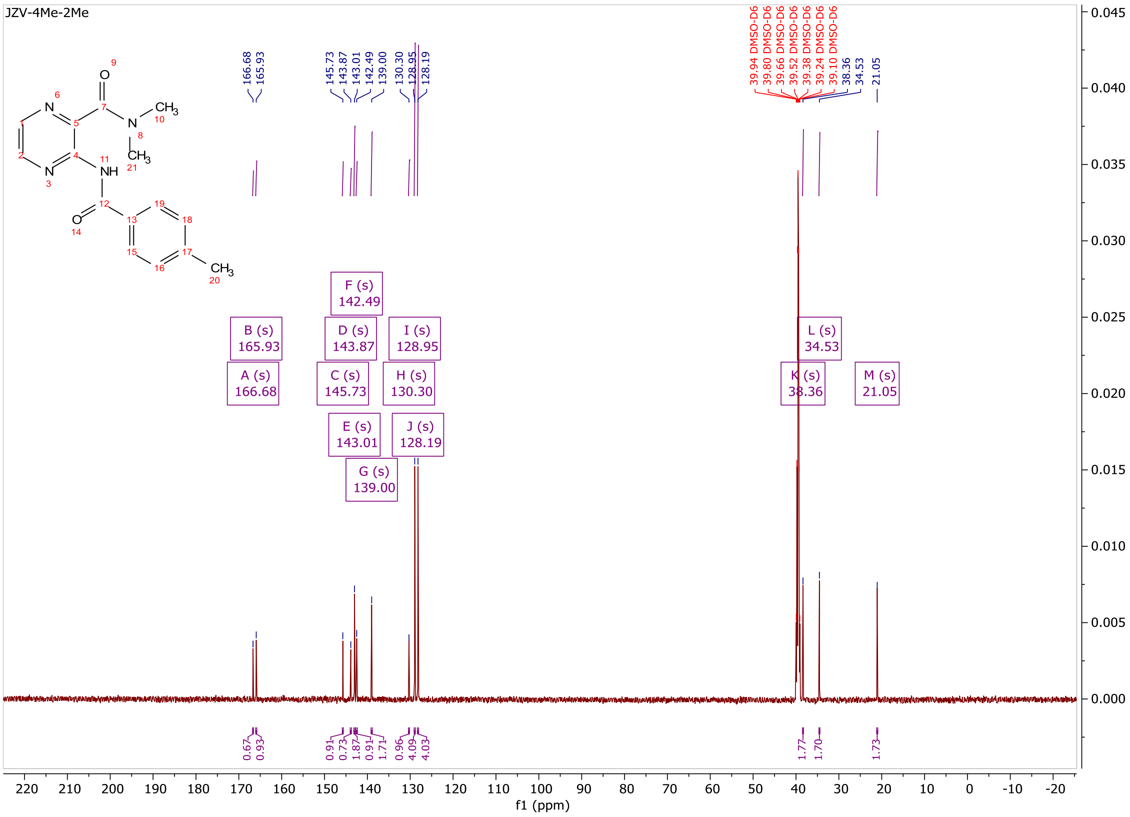

JZV-4Me-2Me
166.68
165.93
145.73
143.87
143.01
142.49
139.00
130.30
128.95
128.19
39.94 DMSO-D6
39.80 DMSO-D6
39.66 DMSO-D6
39.52 DMSO-D6
39.38 DMSO-D6
39.24 DMSO-D6
39.10 DMSO-D6
38.36
34.53
21.05
A (s)
166.68
B (s)
165.93
C (s)
145.73
D (s)
143.87
E (s)
143.01
F (s)
142.49
G (s)
139.00
H (s)
130.30
I (s)
128.95
J (s)
128.19
K (s)
38.36
L (s)
34.53
M (s)
21.05
0.67
0.93
0.91
0.73
1.87
0.91
1.71
0.96
4.09
4.03
1.77
1.70
1.73
f1 (ppm)

Compound **40**

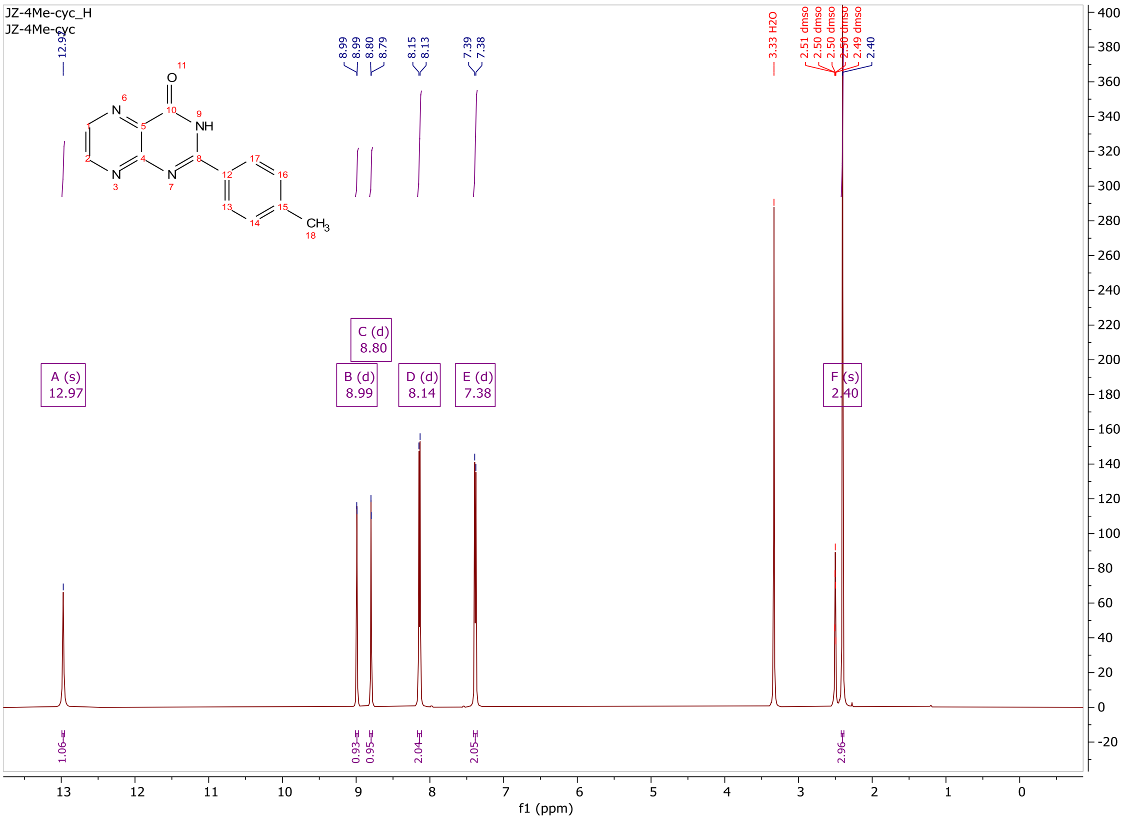
JZ-4Me-cyc_H
JZ-4Me-cyc
12.97
8.99
8.99
8.80
8.79
8.15
8.13
7.39
7.38
3.33 H2O
2.51 dmso
2.50 dmso
2.50 dmso
2.50 dmso
2.49 dmso
2.40
A (s)
12.97
B (d)
8.99
C (d)
8.80
D (d)
8.14
E (d)
7.38
F (s)
2.40
1.06
0.93
0.95
2.04
2.05
2.96
f1 (ppm)

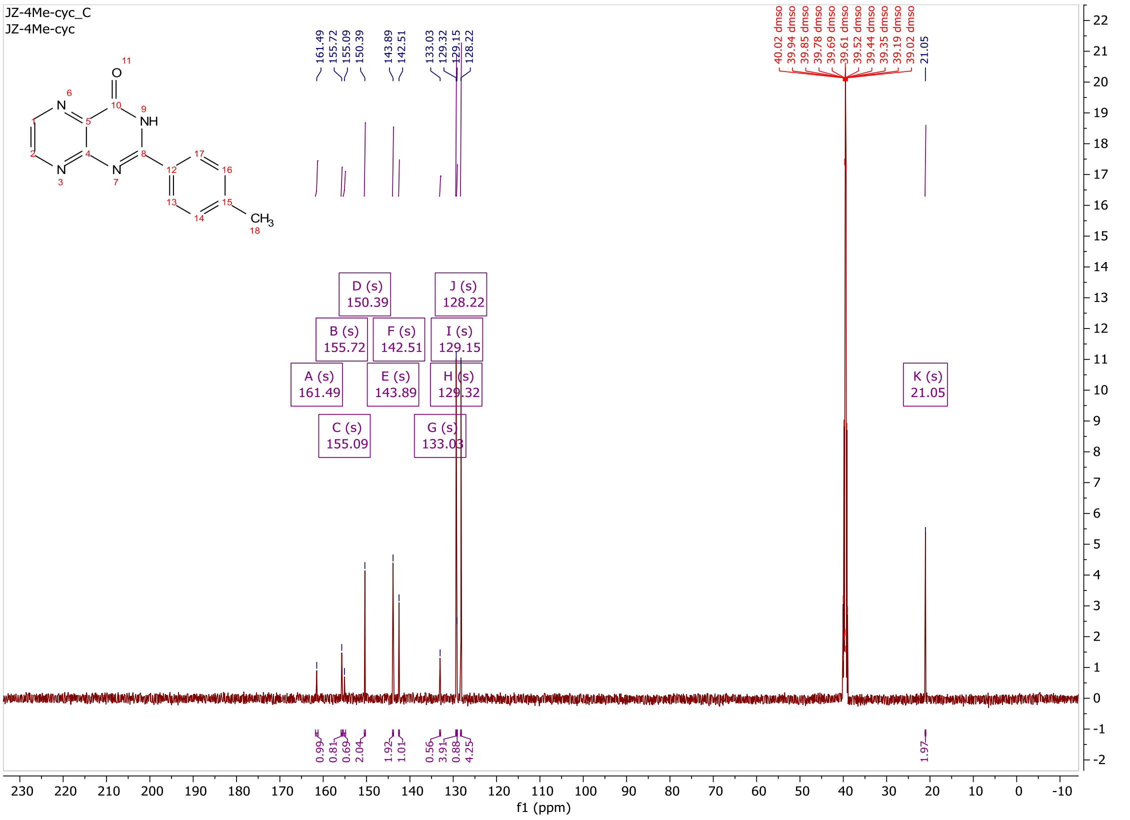
JZ-4Me-cyc_C
JZ-4Me-cyc
161.49
155.72
155.09
150.39
143.89
142.51
133.03
129.32
129.15
128.22
40.02 dmso
39.94 dmso
39.85 dmso
39.78 dmso
39.69 dmso
39.61 dmso
39.52 dmso
39.44 dmso
39.35 dmso
39.19 dmso
39.02 dmso
21.05
A (s)
161.49
B (s)
155.72
C (s)
155.09
D (s)
150.39
E (s)
143.89
F (s)
142.51
G (s)
133.03
H (s)
129.32
I (s)
129.15
J (s)
128.22
K (s)
21.05
f1 (ppm)

Compound **4e** (ester intermediate)

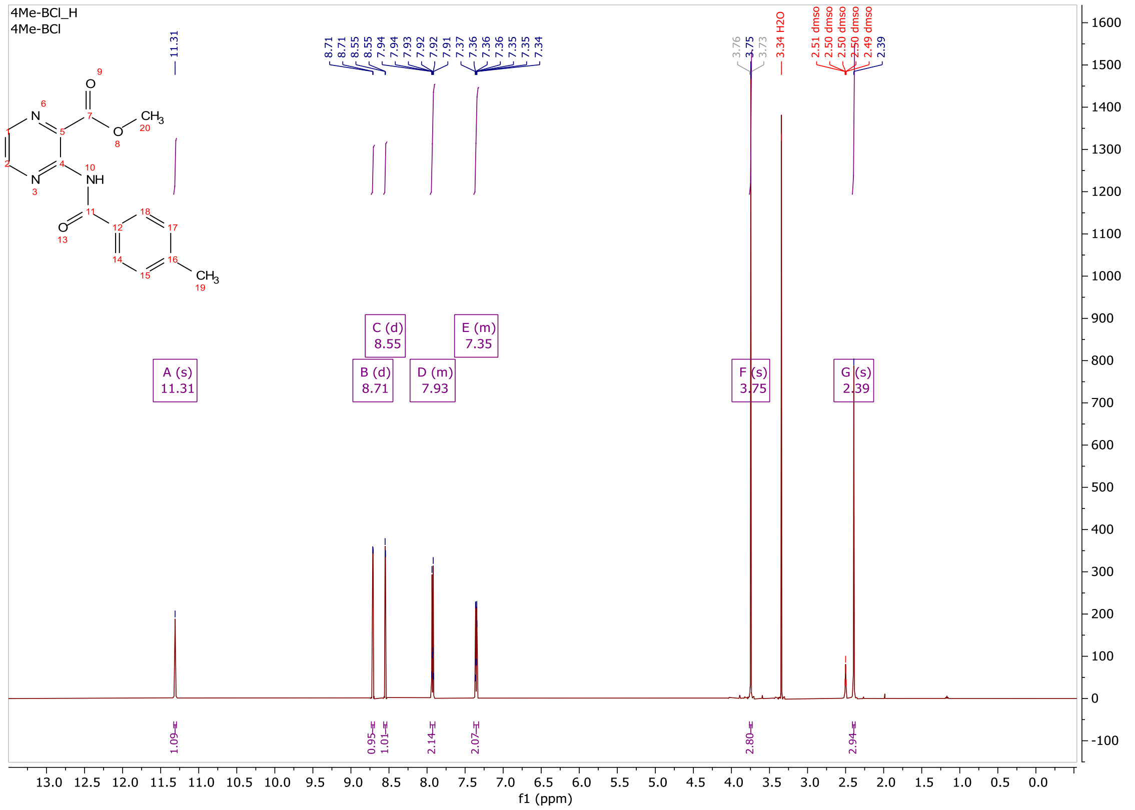

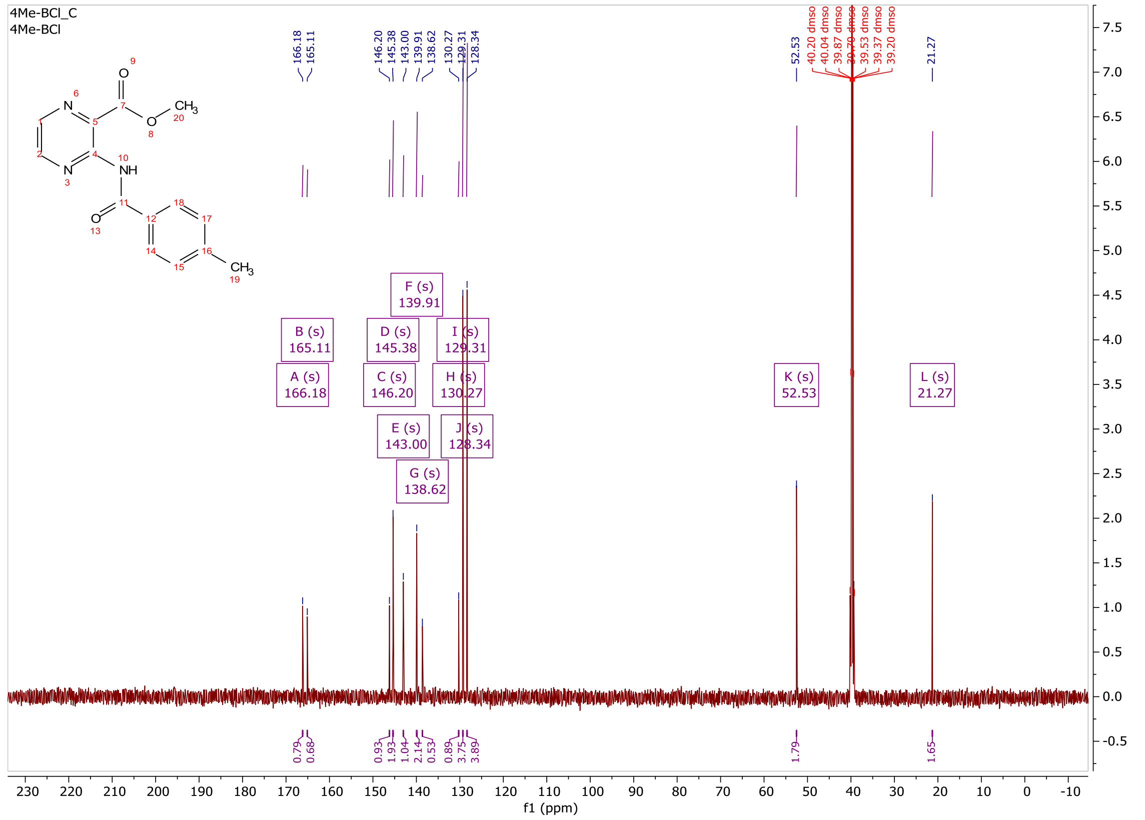

2.3. Results of antimycobacterial activity screening

The antimycobacterial activities of the products are in the main text. We tested some of the intermediates as well. The intermediate ester derivatives are labelled with an additional "e" to the numerical code corresponding to the final product. MIC in µg/ml can be seen in Table S4.

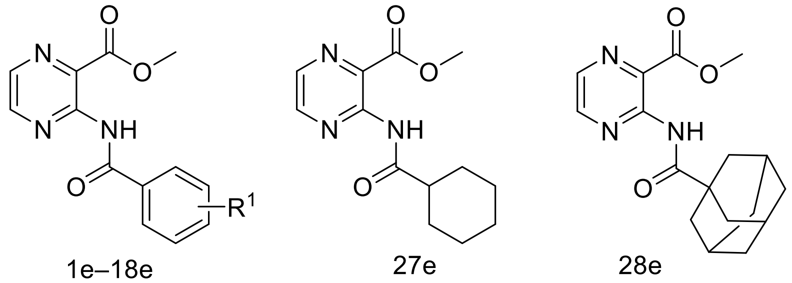

1e–18e 27e 28e

Table S4 *Antimycobacterial activity of methyl ester intermediates*

Code	R[1]	*Mtb* H37Ra MIC [µg/mL]	*M. smegmatis* MIC [µg/mL]	*M. aurum* MIC [µg/mL]	Log P
1e	H	≥500	125	250	0.961
4e	4-Me	250	62.5	250	1.460
9e	4-OMe	≥500	≥500	≥500	1.045
10e	2-F	250	≥500	≥500	0.702
11e	3-F	250	≥500	≥500	1.112
12e	4-F	≥500	≥500	≥500	1.112
13e	2-Cl	≥500	250	≥500	0.852
14e	3-Cl	250	125	250	1.682
16e	2-Br	125	≥500	≥500	0.912
17e	3-Br	≥500	≥500	≥500	1.832
18e	4-Br	≥500	≥500	≥500	1.832
27e	-	≥500	125	≥500	1.503
28e	-	≥125	≥125	≥125	2.131

Log P - calculated from ChemDraw

2.4. Results of antibacterial activity screening

Table S5 *Antibacterial activities against tested bacterial species. MIC read after 24 h, expressed in µM* *(continued on the following pages)*

Code	SA	MRSA	SE	EF	EC	KP	ACI	PA
1	>500	>500	>500	>500	>500	>500	>500	>500
2	>500	>500	>500	>500	>500	>500	>500	>500
3	>500	>500	>500	>500	>500	>500	>500	>500
4	>500	>500	>500	>500	>500	>500	>500	>500
5	>250	>250	>250	>250	>250	>250	>250	>250
6	>125	>125	>125	>125	>125	>125	>125	>125
7	>500	>500	>500	>500	>500	>500	>500	>500
8	>500	>500	>500	>500	>500	>500	>500	>500
9	>125	>125	>125	>125	>125	>125	>125	>125
10	>500	>500	>500	>500	>500	>500	>500	>500
11	>250	>250	>250	>250	>250	>250	>250	>250
12	>500	>500	>500	>500	>500	>500	>500	>500
13	>500	>500	>500	>500	>500	>500	>500	>500
14	>125	>125	>125	>125	>125	>125	>125	>125
15	>500	>500	>500	>500	>500	>500	>500	>500
16	>500	>500	>500	>500	>500	>500	>500	>500
17	>125	>125	>125	>125	>125	>125	>125	>125
18	>500	>500	>500	>500	>500	>500	>500	>500
19	>500	>500	>500	>500	>500	>500	>500	>500
20	>500	>500	>500	>500	>500	>500	>500	>500
21	>125	>125	>125	>125	>125	>125	>125	>125
22	n.d	n.d	n.d	n.d	n.d	n.d	n.d	n.d
23	n.d	n.d	n.d	n.d	n.d	n.d	n.d	n.d
24	n.d	n.d	n.d	n.d	n.d	n.d	n.d	n.d
25	>250	>250	>250	>250	>250	>250	>250	>250
26	>500	>500	>500	>500	>500	>500	>500	>500
27	>500	>500	>500	>500	>500	>500	>500	>500
28	>500	>500	>500	>500	>500	>500	>500	>500
29	>500	>500	>500	>500	>500	>500	>500	>500
30	>500	>500	>500	>500	>500	>500	>500	>500
31	>125	>125	>125	>125	>125	>125	>125	>125
32	>250	>250	>250	>250	>250	>250	>250	>250
33	>500	>500	>500	>500	>500	>500	>500	>500
34	>125	>125	>125	>125	>125	>125	>125	>125
35	>125	>125	>125	>125	>125	>125	>125	>125
36	>125	>125	>125	>125	>125	>125	>125	>125
37	>500	>500	>500	>500	>500	>500	>500	>500
38	n.d	n.d	n.d	n.d	n.d	n.d	n.d	n.d
39	>125	>125	>125	>125	>125	>125	>125	>125
40	>125	>125	>125	>125	>125	>125	>125	>125
41	>125	>125	>125	>125	>125	>125	>125	>125
42	>125	>125	>125	>125	>125	>125	>125	>125
43	>125	>125	>125	>125	>125	>125	>125	>125
1e	>500	>500	>500	>500	>500	>500	>500	>500
4e	>500	>500	>500	>500	>500	>500	>500	>500
9e	>500	>500	>500	>500	>500	>500	>500	>500
10e	n.d	n.d	n.d	n.d	n.d	n.d	n.d	n.d

11e	n.d	n.d	n.d	n.d	n.d	n.d	n.d	n.d
12e	n.d	n.d	n.d	n.d	n.d	n.d	n.d	n.d
13e	n.d	n.d	n.d	n.d	n.d	n.d	n.d	n.d
14e	>500	>500	>500	>500	>500	>500	>500	>500
16e	>500	>500	>500	>500	>500	>500	>500	>500
17e	>500	>500	>500	>500	>500	>500	>500	>500
18e	>500	>500	>500	>500	>500	>500	>500	>500
27e	>500	>500	>500	>500	>500	>500	>500	>500
28e	>125	>125	>125	>125	>125	>125	>125	>125

SA - *Staphylococcus aureus* subsp. *aureus* CCM 4223 (ATCC 29213); MRSA - *Staphylococcus aureus* subsp. *aureus* (MRSA) CCM 4750 (ATCC 43300); SE - *Staphylococcus epidermidis* CCM 4418 (ATCC 12228); EF - *Enterococcus faecalis* CCM 4224 (ATCC 29212); EC - *Escherichia coli* CCM 3954 (ATCC 25922); KP - *Klebsiella pneumoniae* CCM 4415 (ATCC 10031); AB - *Acinetobacter baumannii* DSM 30007 (ATCC 19606); PA - *Pseudomonas aeruginosa* CCM 3955 (ATCC 27853); n.d. – not determined. No significant change in the activities was observed after 48 h incubation (data not enclosed).

2.5. Results of antifungal screening

Table S6 *Antifungal activities against tested fungal species. MIC read after 24 h (72 h for TI), expressed in µM (continued on the following pages).*

Code	CA	CK	CP	CT	AF	AFla	LC	TI
1	>500	>500	>500	>500	>500	>500	>500	>500
2	>125	>125	>125	>125	>125	>125	>125	>125
3	>500	>500	>500	>500	>500	>500	>500	>500
4	>500	>500	>500	>500	>500	>500	>500	>500
5	>250	>250	>250	>250	>250	>250	>250	>250
6	>125	>125	>125	>125	>125	>125	>125	>125
7	>500	>500	>500	>500	>500	>500	>500	>500
8	>500	>500	>500	>500	>500	>500	>500	>500
9	>125	>125	>125	>125	>125	>125	>125	>125
10	>500	>500	>500	>500	>500	>500	>500	>500
11	>250	>250	>250	>250	>250	>250	>250	>250
12	>500	>500	>500	>500	>500	>500	>500	>500
13	>500	>500	>500	>500	>500	>500	>500	>500
14	>125	>125	>125	>125	>125	>125	>125	>125
15	>125	>125	>125	>125	>125	>125	>125	>125
16	>500	>500	>500	>500	>500	>500	>500	>500
17	>125	>125	>125	>125	>125	>125	>125	>125
18	>125	>125	>125	>125	>125	>125	>125	>125
19	>500	>500	>500	>500	>500	>500	>500	>500
20	>500	>500	>500	>500	>500	>500	>500	>500
21	>125	>125	>125	>125	>125	>125	>125	>125
22	n.d	n.d	n.d	n.d	n.d	n.d	n.d	n.d
23	n.d	n.d	n.d	n.d	n.d	n.d	n.d	n.d
24	n.d	n.d	n.d	n.d	n.d	n.d	n.d	n.d
25	>250	>250	>250	>250	>250	>250	>250	>250
26	>500	>500	>500	>500	>500	>500	>500	>500
27	>500	>500	>500	>500	>500	>500	>500	>500
28	500	500	>500	>500	>500	>500	>500	125
29	>500	>500	>500	>500	>500	>500	>500	>500

30	>500	>500	>500	>500	>500	>500	>500	>500
31	>125	>125	>125	>125	>125	>125	>125	>125
32	>250	>250	>250	>250	>250	>250	>250	>250
33	>500	>500	>500	>500	>500	>500	>500	>500
34	>125	>125	>125	>125	>125	>125	>125	>125
35	>125	>125	>125	>125	>125	>125	>125	>125
36	>125	>125	>125	>125	>125	>125	>125	>125
37	>500	>500	>500	>500	>500	>500	>500	>500
38	n.d	n.d	n.d	n.d	n.d	n.d	n.d	n.d
39	>125	>125	>125	>125	>125	>125	>125	>125
40	>125	>125	>125	>125	>125	>125	>125	>125
41	>125	>125	>125	>125	>125	>125	>125	>125
42	>125	>125	>125	>125	>125	>125	>125	>125
43	>125	>125	>125	>125	>125	>125	>125	>125
1e	>500	>500	>500	>500	>500	>500	>500	>500
4e	>500	>500	>500	>500	>500	>500	>500	>500
9e	>500	>500	>500	>500	>500	>500	>500	>500
10e	n.d	n.d	n.d	n.d	n.d	n.d	n.d	n.d
11e	n.d	n.d	n.d	n.d	n.d	n.d	n.d	n.d
12e	n.d	n.d	n.d	n.d	n.d	n.d	n.d	n.d
13e	n.d	n.d	n.d	n.d	n.d	n.d	n.d	n.d
14e	>500	>500	>500	>500	>500	>500	>500	>500
16e	>500	>500	>500	>500	>500	>500	>500	>500
17e	>500	>500	>500	>500	>500	>500	>500	>500
18e	>500	>500	>500	>500	>500	>500	>500	>500
27e	>500	>500	>500	>500	>500	>500	>500	>500
28e	>125	>125	>125	>125	>125	>125	>125	>125

CA - *Candida albicans* CCM 8320 (ATCC 24433); CK - *Candida krusei* CCM 8271 (ATCC 6258); CP - *Candida parapsilosis* CCM 8260 (ATCC 22019); CT - *Candida tropicalis* CCM 8264 (ATCC 750); AF - A*spergillus fumigatus* (ATCC 204305); AFla - *Aspergillus flavus* CCM 8363; LC - *Lichtheimia corymbifera* CCM 8077; TI - *Trichophyton interdigitale* CCM 8377 (ATCC 9533); n.d. – not determined. No significant change in the activities was observed after 48 h (72 h for TI) incubation (data not enclosed).

2.6. Results for HepG2 cytotoxicity

Representative IC_{50} curves:

Comound **5**
IC_{50} > 100 µM

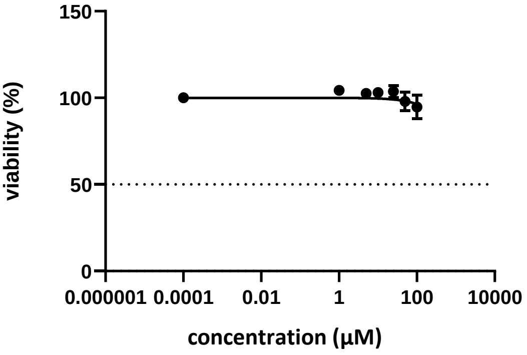

Compound **14**
IC_{50} > 25 µM

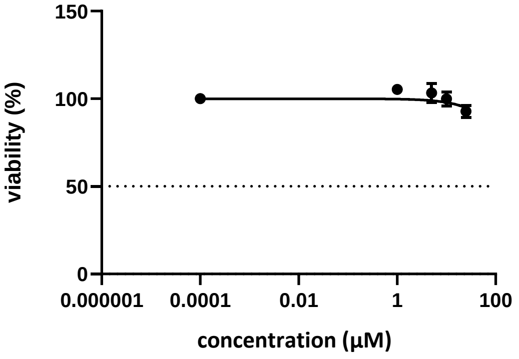

Compound **21**
IC_{50} > 100 µM

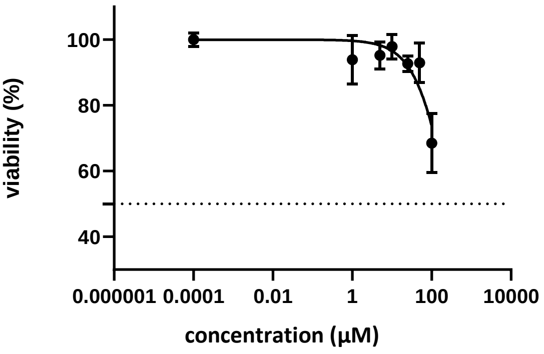

Compound **23**
IC_{50} > 500 µM

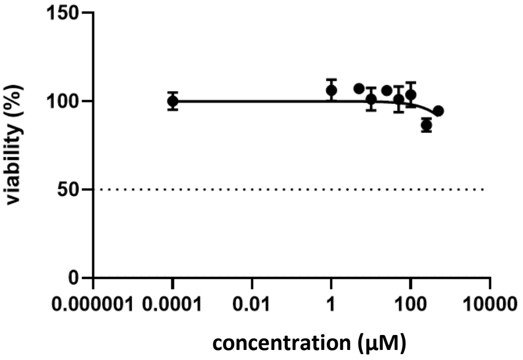

Compound **24**
IC_{50} = 929.0 µM

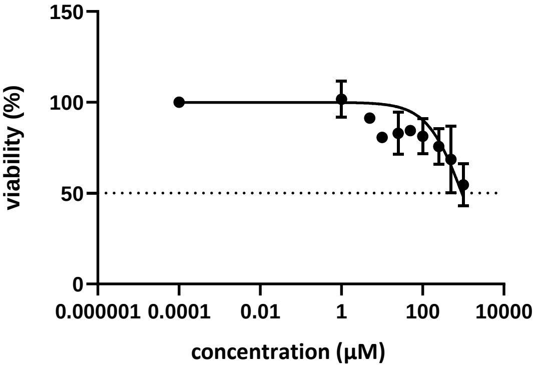

Compound **30**
IC_{50} = 560.3 µM

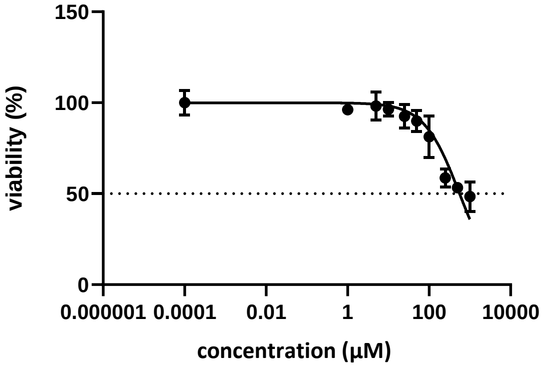

Compound **33**
IC_{50} = 764.7 µM

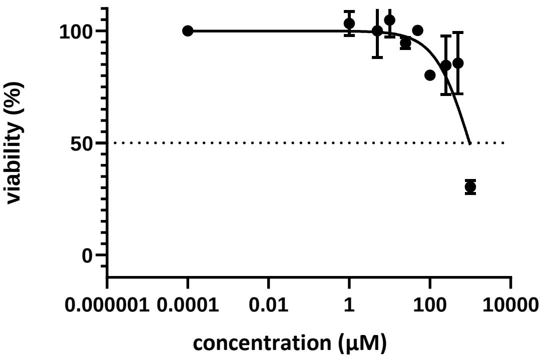

Compound **36**
IC_{50} > 1000 µM

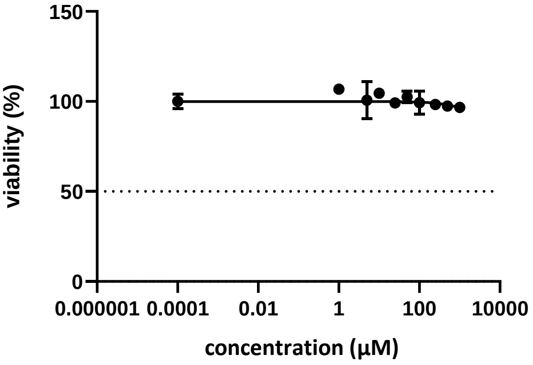

Compound **38** (Adachi)
IC_{50} = 19.6 µM

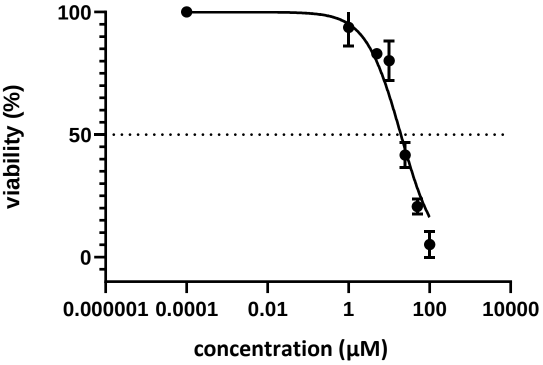

Compound **39**
IC_{50} > 1000 µM

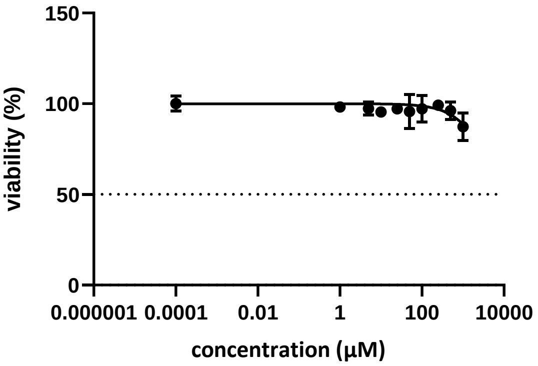

Compound **40**
IC_{50} > 1000 µM

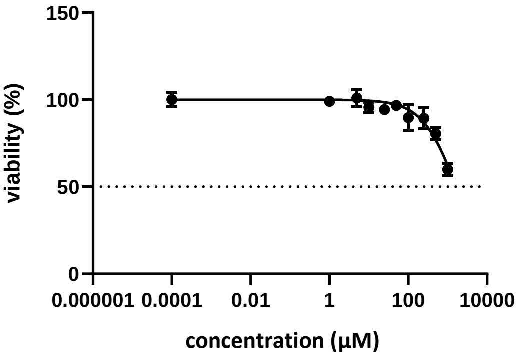

2.7. Results of *in silico* simulations

Figure S1 presents the results of the induced fit docking of compound **15** to the homology model of mtProRS. This pose was then taken as a starting system for molecular dynamics (MD), see below. The binding mode of **15** was consistent of the binding mode of adenosine (Figure S1, panel C).

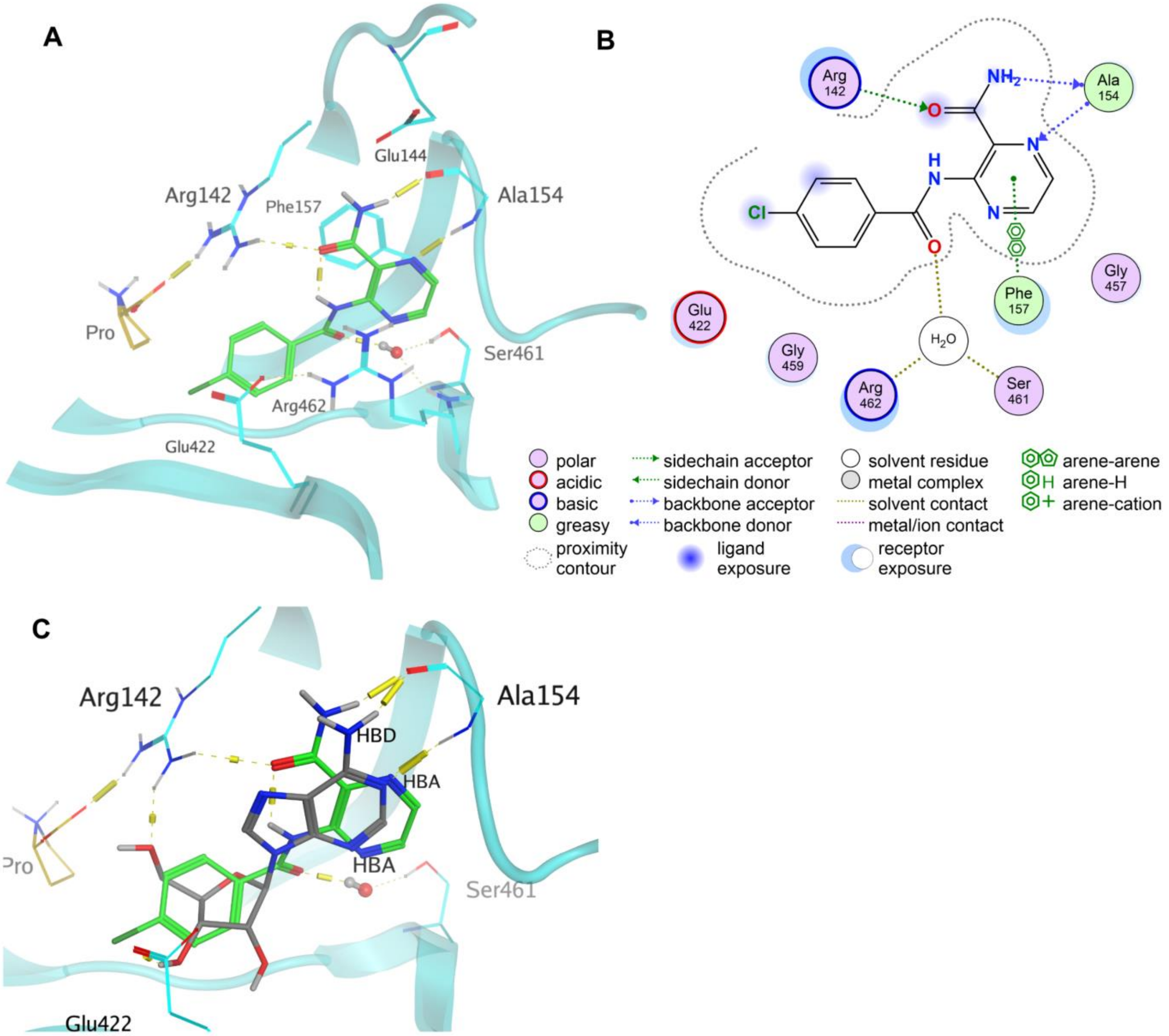

Figure S1. Compound **15** docked (induced fit docking) into the homology model of mtProRS. A – Ligand-receptor interactions in 3D; B – 2D ligand-receptor interaction diagram; C – detail of the overlay of the docked pose of compound **15** with adenosine

Figure S2 presents the additional analysis of the MD simulation of the ternary complex of compound **15** and proline in mycobacterial ProRS. Three independent production runs of 50 ns were performed. Panels **A** and **B** show stabilisation of both protein (central domain) and ligand **15**. The low movement of ligand **15** is also documented by low RMSF values (panel **C**) for individual atoms of the ligand, where most of the heavy atoms had RMSF < 0.8 Å. Increased RMSF for the benzene carbons C2', C3', C5' and C6' (annotation from the MD system is C16, C15, C19 and C18, respectively, see panel **D**), with the concomitant low RMSF of the Cl atom, and C4' (C13) and C1' (C17) of the benzene ring, indicate the partial rotation of around the C1'-C4'-Cl axis of the benzene ring.

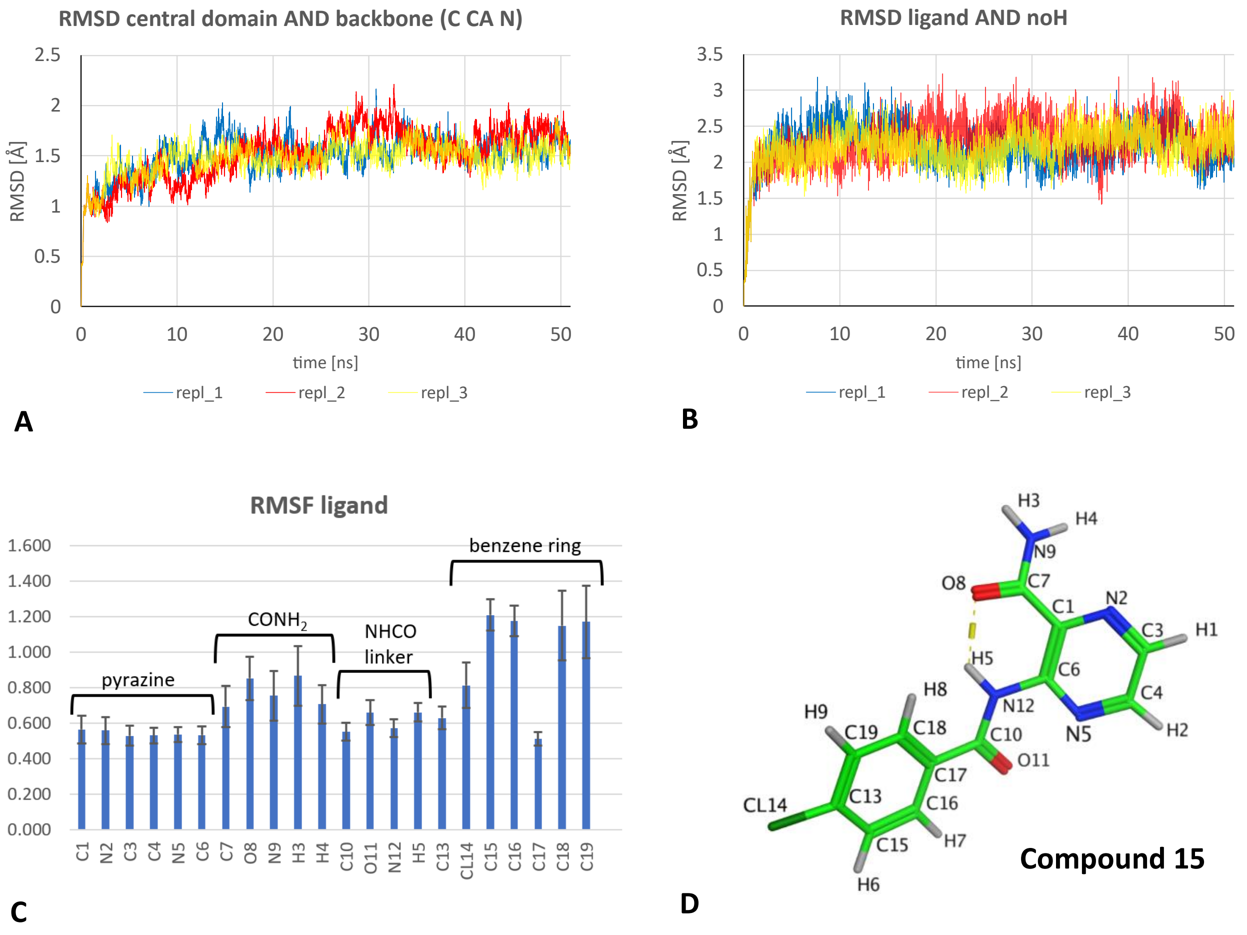

Figure S2. Results of trajectory analysis of MD simulation of the ternary complex of ligand **15** and proline cofactor in mtProRS. A – RMSD of the central domain backbone; B – RMSD of ligand (heavy atoms); C – RMSF (in Å) on individual ligand atoms; D – labelling of individual atoms of the ligand

Figure S3 analyses the distance between the chlorine atom of ligand **15** and both carboxylate oxygen atoms of Glu211 sidechain. The original Cl-O distance in the docked pose (used as a starting point for MD, Figure S1) was about 8 Å, but dropped to 4 Å during the equilibration phase (towards the end of the first ns), and then remained stable until the end of the simulation. The occasional swapping of the red and blue lines (annotated by bald arrows) indicated the mutual exchange of positions of the equivalent oxygen atoms of the carboxylate.

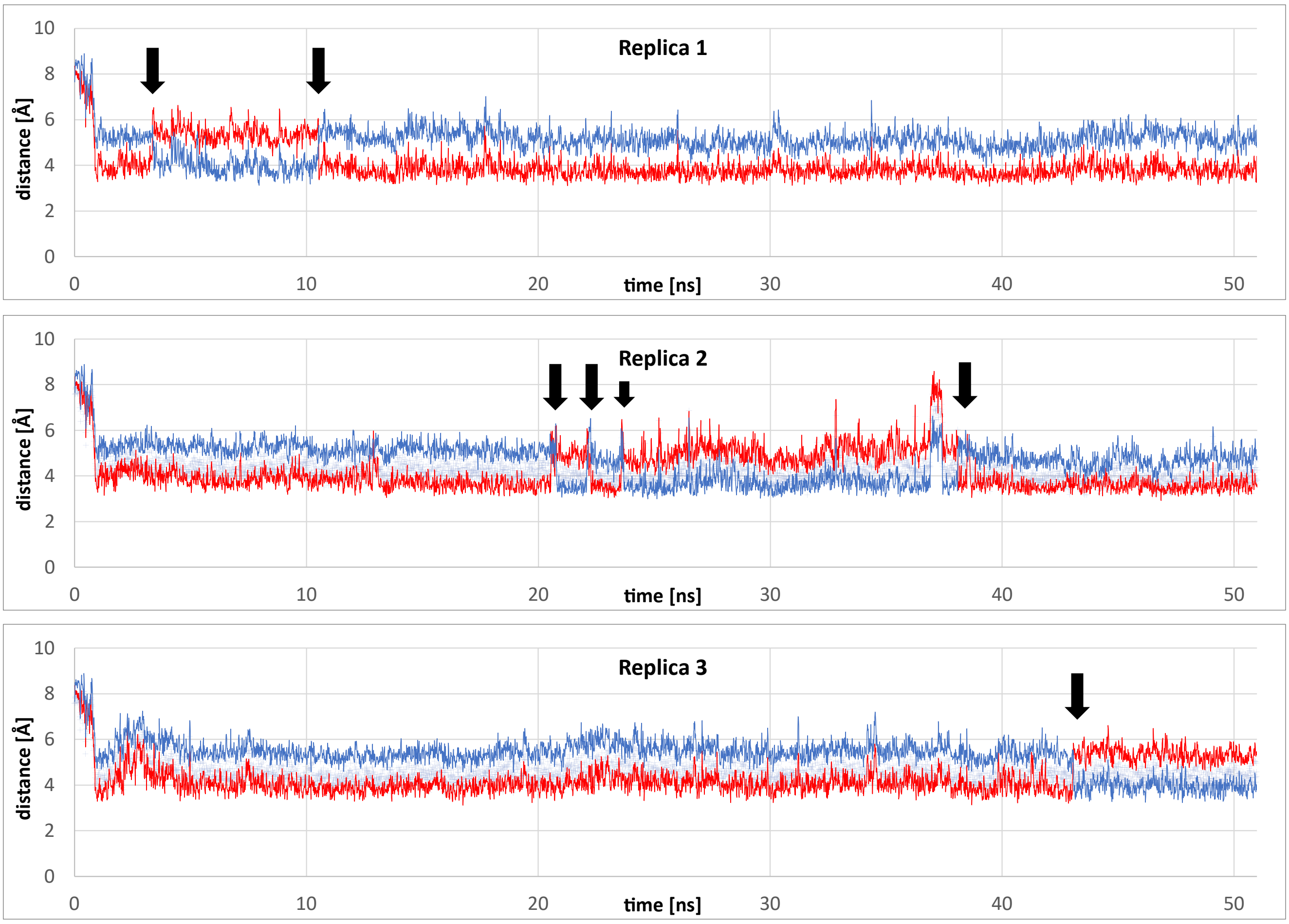

Figure S3. Distance between Cl atom of ligand **15** and two carboxylate oxygen atoms of Glu211, along the MD trajectories.

Figure S4 presents the additional analysis of MD simulations of the ternary complex of compound **34** and proline in mycobacterial ProRS. Three independent production runs of 50 ns were performed. The stabilisation of the ligand, as indicated by RMSD curves in panel B, was significantly worse compared to ligand **15**. the RMSF values for individual atoms of the ligand (panel **B**) were in general 0.2 Å higher compared to ligand **15**.

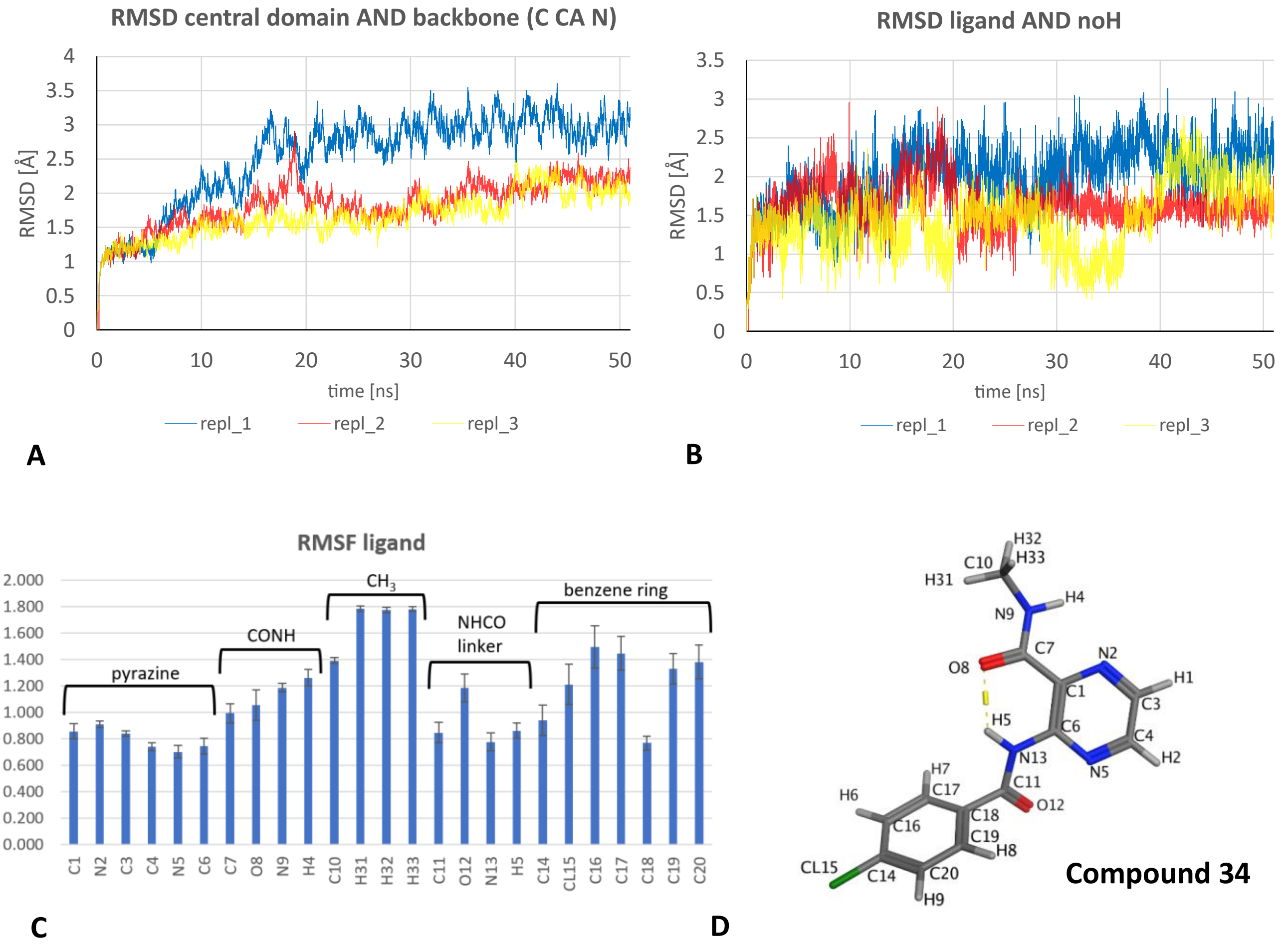

Figure S4. Results of trajectory analysis of MD simulation of the ternary complex of ligand **34** and proline cofactor in mtProRS. A – RMSD of the central domain backbone; B – RMSD of ligand (heavy atoms); C – RMSF (in Å) on individual ligand atoms; D – labelling of individual atoms of the ligand

In contrast to the simulation of ligand **15**, the chlorine atom of compound **34** did not come closer than 6Å to the oxygen atoms of the carboxylate of Glu211 (Fig. S5).

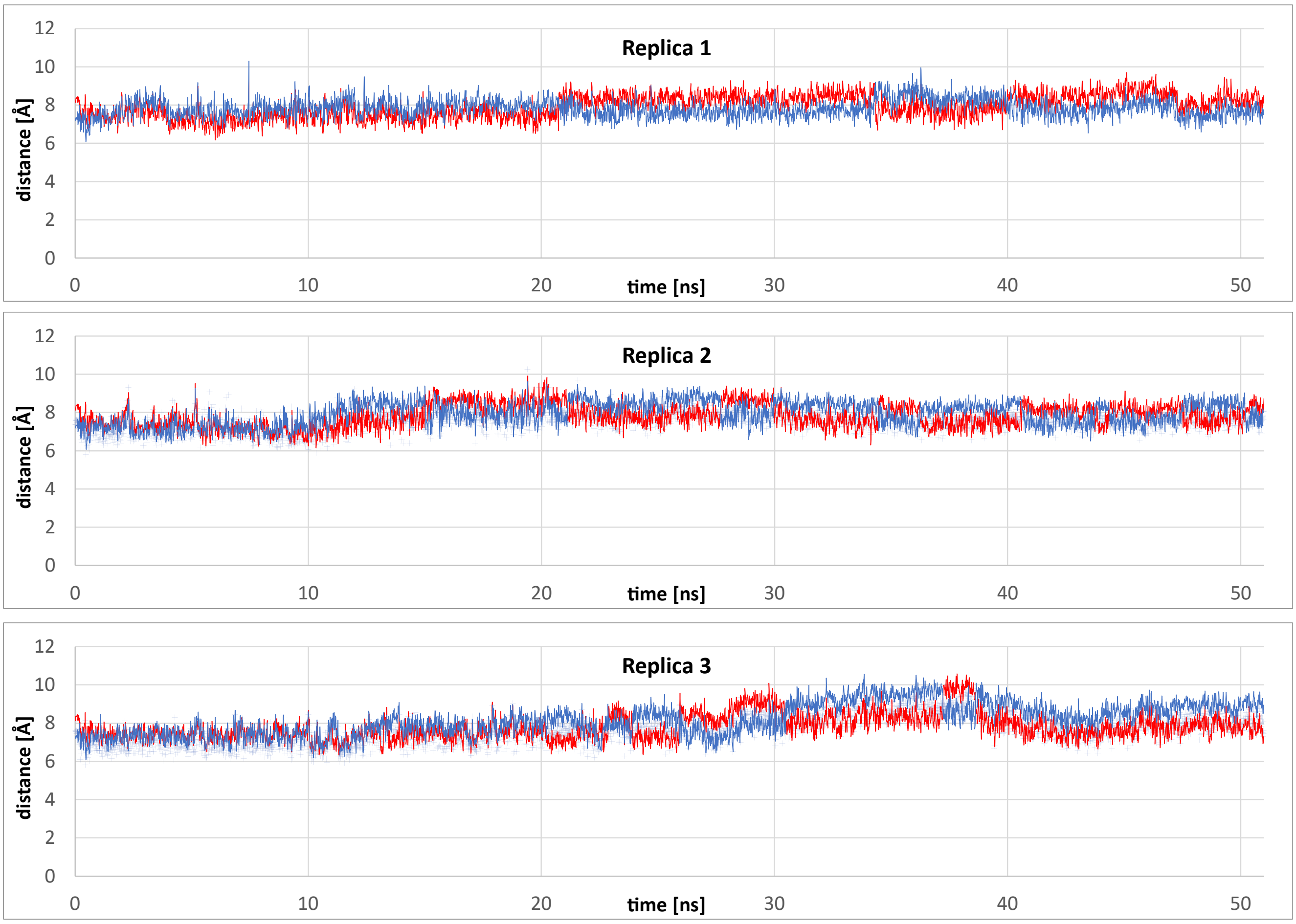

Figure S5. Distance between Cl atom of ligand **34** and two carboxylate oxygen atoms of Glu211, along the MD trajectories.

3. References

(1) European Committee for Antimicrobial Susceptibility Testing (EUCAST) of the European Society for Clinical Microbiology and Infectious Diseases (ESCMID). EUCAST Discussion Document E. Dis 5.1: determination of minimum inhibitory concentrations (MICs) of antibacterial agents by broth dilution. Clin Microbiol Infec 2003; 9:1-7. http://www.eucast.org/documents/publications_in_journals/ (Accessed 11 Dec, 2019).

(2) EUCAST DEFINITIVE DOCUMENT E.DEF 7.3.1. Method for the determination of broth dilution minimum inhibitory concentrations of antifungal agents for yeasts. 2017. http://www.eucast.org/astoffungi/methodsinantifungalsusceptibilitytesting/susceptibility_testing_of_yeasts/ (Accessed 11 Dec, 2019).

(3) EUCAST DEFINITIVE DOCUMENT E.DEF 9.3.1. Method for the determination of broth dilution minimum inhibitory concentrations of antifungal agents for conidia forming moulds. 2017. http://www.eucast.org/astoffungi/methodsinantifungalsusceptibilitytesting/susceptibility_testing_of_moulds/ (Accessed 11 Dec, 2019).
